# Suppression of inflammatory and infection responses in lung macrophages by eucalyptus oil and its constituent 1,8-cineole: Role of pattern recognition receptors TREM-1 and NLRP3, the MAP kinase regulator MKP-1, and NFκB

**DOI:** 10.1371/journal.pone.0188232

**Published:** 2017-11-15

**Authors:** Niket Yadav, Harish Chandra

**Affiliations:** Microbial Pathogenesis and Immunotoxicology Laboratory, University of Cincinnati College of Medicine, Cincinnati, Ohio, United States of America; University of Texas Medical Branch at Galveston, UNITED STATES

## Abstract

Eucalyptus oil (EO) used in traditional medicine continues to prove useful for aroma therapy in respiratory ailments; however, there is a paucity of information on its mechanism of action and active components. In this direction, we investigated EO and its dominant constituent 1,8–cineole (eucalyptol) using the murine lung alveolar macrophage (AM) cell line MH-S. In an LPS-induced AM inflammation model, pre-treatment with EO significantly reduced (P ≤0.01or 0.05) the pro-inflammatory mediators TNF-α, IL-1 (α and β), and NO, albeit at a variable rate and extent; 1,8-cineole diminished IL-1 and IL-6. In a mycobacterial-infection AM model, EO pre-treatment or post-treatment significantly enhanced (P ≤0.01) the phagocytic activity and pathogen clearance. 1,8-cineole also significantly enhanced the pathogen clearance though the phagocytic activity was not significantly altered. EO or 1,8-cineole pre-treatment attenuated LPS-induced inflammatory signaling pathways at various levels accompanied by diminished inflammatory response. Among the pattern recognition receptors (PRRs) involved in LPS signaling, the TREM pathway surface receptor (TREM-1) was significantly downregulated. Importantly, the pre-treatments significantly downregulated (P ≤0.01) the intracellular PRR receptor NLRP3 of the inflammasome, which is consistent with the decrease in IL-1β secretion. Of the shared downstream signaling cascade for these PRR pathways, there was significant attenuation of phosphorylation of the transcription factor NF-κB and p38 (but increased phosphorylation of the other two MAP kinases, ERK1/2 and JNK1/2). 1,8-cineole showed a similar general trend except for an opposite effect on NF-κB and JNK1/2. In this context, either pre-treatment caused a significant downregulation of MKP-1 phosphatase, a negative regulator of MAPKs. Collectively, our results demonstrate that the anti-inflammatory activity of EO and 1,8-cineole is modulated via selective downregulation of the PRR pathways, including PRR receptors (TREM-1 and NLRP3) and common downstream signaling cascade partners (NF-κB, MAPKs, MKP-1). To our knowledge, this is the first report on the modulatory role of TREM-1 and NLRP3 inflammasome pathways and the MAPK negative regulator MKP-1 in context of the anti-inflammatory potential of EO and its constituent 1,8-cineole.

## Introduction

The growing prevalence of infection-associated and other inflammatory conditions and diseases in modern healthcare necessitates novel therapeutic interventions. Synthetic steroidal and non-steroidal anti-inflammatories and antibiotics commonly used for treating inflammation and infection conditions often result in undesirable side effects and health consequences [1]. This has evoked renewed global interest for alternative safe therapeutics from natural sources. Natural products continue to inspire the design of novel therapeutics for various disease conditions; however, little is known regarding their mode of action and active components.

Different traditional medicinal systems such as Indian Ayurveda and Yunani medicines have been using natural oils and extracts from plants to heal infections and other ailments [2–4]. One of the important medicinal plant products is eucalyptus oil (EO) derived from *Eucalyptus globulus* belonging to the family Myrtaceae, of which several species are now found throughout the world [5]. EO has been widely used in treatment of upper respiratory conditions [5], and other ailments such as gastritis, diabetes and pain associated with total knee replacement [6–7]. Independent studies have reported analgesic and anti-inflammatory properties of EO and its major component 1, 8- cineole, which accounts for up to more than 70% of eucalyptus oil’s content depending on the source species of Eucalyptus [8–11].

In vitro studies by us [12] and others have shown EO to be antimicrobial, including effective against respiratory pathogens [13]. One of the striking examples is the use of EO in aroma therapy to heal lung infections such as tuberculosis in traditional medicine in Africa and other continents [2, 14]. Additionally, EO has been reported to be effective against multidrug resistant strains such as methicillin-resistant *Staphylococcus aureus* [15] and biofilm-forming *Staphylococcus aureus* and *pseudomonas aeruginosa* strains [16].

In light of the above extraordinarily broad in vitro anti-inflammatory and antimicrobial activities of EO and its traditional use in aroma therapy, we wanted to investigate the mechanistic basis of these therapeutic effects using a lung immune cell model. Alveolar macrophages (AMs) are one of the first line of innate defenses in the lungs and are known to play a significant role in inflammation and respiratory infections, including mycobacterial infections such as tuberculosis [17–18]. Hence AMs are appropriate target cells to study the effect of eucalyptus oil on the lungs. The current study employed the mouse alveolar macrophage cell line MH-S as the lung macrophage model, LPS as the inducer of the inflammatory response (a bacterial virulence factor in Gram-negative infection conditions such as sepsis), and *M*. *smegmatis* as the model Mycobacterium *species* capable of infecting alveolar macrophages, to investigate the immunomodulatory effects and mode of action of EO and its constituent 1,8-cineole. Mechanistic aspects of the study focused on investigating modulation of LPS-induced inflammation signaling pathways by measuring transcriptional or post-translational modification of multiple key upstream and downstream signaling receptors and cascades, yielding novel insights on modes of action of EO and its constituent 1,8-cineole.

## Materials and methods

### Natural products, chemicals, reagents, and kits

Eucalyptus oil (EO) originally derived from *Eucalyptus globulus* and was supplied by Ashwin Pharma, MH, India, purchased via the local vendor Bombay Grocers, Cincinnati, OH, USA; the working stock (2% vol/vol) was prepared in 10% methanol (vehicle). Purified EO constituent 1,8-cineole (eucalyptol) was obtained commercially (Alfa Aesar, Reston, VA, USA); the working stock (2% vol/vol or 0.119 M) was prepared in 10% methanol (MeOH). The bacterial lipopolysaccharide (LPS) was obtained commercially (Sigma, St. Louis, MO, USA) and the working stocks (1 mg/ml and 0.2 mg/ml) were made by dissolving in endotoxin-free water. Anti-MAPK antibodies, both total and phospho antibodies raised in rabbit (Cell Signaling Technology, Danvers, MA, USA), secondary horseradish peroxidase (HRP)-conjugated goat antibodies (Sigma, USA), and anti β-Actin mouse monoclonal antibodies (Sigma, USA) were obtained commercially. Cytokine ELISA kits were obtained from Affymetrix Inc. eBioscience San Diego, CA, USA. All primers were ordered from Integrated DNA technologies (Coralville, IA, USA) and are listed in Table 1. Quantitative RT-PCR kit QRT-PCR Brilliant III Ultra-fast SYBR^®^ green master mix kit was obtained from Agilent Technologies, USA.

**Table 1:** Gene-specific primers for qRT-PCR analysis in this study.

Target gene	Primer Names	Primer Sequences	References
MKP-1	Forward	5’TAACCACTTTGAGGGTCACTACC- 3’	[34]
	Reverse	5’- TTCACAAACTCAAAGGCCTCG- 3’	[34]
mTREM-1	Forward	5’- CCAGAAGGCTTGGCAGAGACT- 3’	[35]
	Reverse:	5’-ACTTCCCCATGTGGACTTCACT-3’	[35]
GAPDH	Forward	5’- ATTGTGGAAGGGCTCATGACC-3’	[34]
	Reverse	5’-ATACTTGGCAGGTTTCTCCAGG-3’	[34]
CD-14	Forward	5’-AGAATCTACCGACCATGGAGC-3’	This study
	Reverse	5’-TGAAAGCGCTGGACCAATCT-3’	This study
TLR4	Forward	5’-TCCCTGCATAGAGGTAGTTCC-3’	This study
	Reverse	5’-TCCAGCCACTGAAGTTCTGA-3’	This study
LBP	Forward	5’-TCCAGACTCTGCCAGTCACA-3’	This study
	Reverse	5’-CTCAGGTAGGCTCATGGTCG-3’	This study

### Strains, cell lines, and culture conditions

For infection studies, the fast-growing model non-tuberculous mycobacterial strain *Mycobacterium smegmatis mc*^*2*^ *155* obtained from American Type Culture Collection (ATCC), Manassas, VA, USA was used. It was grown in Sauton’s medium, containing L-asparagine (26mM), citric acid (10mM), K_2_HPO_4_ (2mM), MgSO_4_.7H_2_O (2mM), ferric ammonium citrate (0.18 mM), tween 80 (0.05%) and glycerol (0.2%), at 37°C in a shaker incubator (200 rpm). Actively growing mycobacterial cells were harvested by centrifugation, washed, and resuspended in RPMI medium (1x 10^7^ CFU/ml) for use as inoculum for all infection experiments. A murine alveolar macrophage cell line MH-S (CRL-2019) from ATCC (Manassas, VA) was used. MH-S cells were grown at 37°C in a humidified 5% CO_2_ incubator in either complete RPMI 1640 medium supplemented with 10% Hyclone fetal bovine serum (Logan, UT, USA) and 1x streptomycin-penicillin-glutamate antibiotic solution (Gibco, NY, USA) or incomplete RPMI medium without antibiotic for the purpose of phagocytosis experiments. MH-S cells were harvested using a sterile cell scraper in fresh RPMI medium and were counted using a hemocytometer and cell concentration was adjusted (1 x 10^6^ cells/ml) in the RPMI medium.

### Selection of optimal concentration of eucalyptus oil (EO) and its constituent 1,8-cineole

MH-S cells seeded at a density of 1×10^6^ cells/ml culture medium were dispensed in 5-ml volumes in multiple T25 flasks (Corning, NY, USA). Varying concentrations of EO or 1,8- cineole (0.0, 0.015, 0.020. 0.050, and 0.100% vol/vol) were added to the flasks to investigate dose-response in terms of macrophage viability. The vehicle (10% methanol) amount did not exceed the non-inhibitory levels (10μl/ml culture i.e. 0.1% vol/vol). Three independent treatments were performed and analyzed.

### LPS challenge

MH-S cells were seeded at a density of 1×10^6^ cells/ml/well and treated at 37°C for different time points (6, 12, and 24 hours) in triplicate wells in 24-well culture cluster plates. Control wells received only vehicle (0.1% MeOH). In the treatment wells, the cells were treated with EO alone (0.02% vol/vol) or LPS alone (2μg/ml) or a combination of EO (0.02%) and LPS (2μg/ml). The combined treatment (EO + LPS) involved a pre-treatment with EO for the first 3 hours followed by addition of LPS at 2μg/ml and further incubation for a time course (total 6, 12, and 24 hours). In the LPS alone set, the LPS treatment of the cells was started at a 3 hour time-point to match it with the combination treatment. For 1,8-cineole experiments, the same treatment design as described above for EO was repeated. The cell-free supernatants from all experiments were collected and analyzed for inflammatory soluble mediators (cytokines and nitric oxide). For understanding early regulation of LPS-induced inflammatory pathways, the LPS treatment lasted for 30 minutes for all signaling targets except NLRP3 (4 hours) following the initial 3 hour pre-treatment with either EO or 1, 8-cineole.

### Phagocytosis activity and intracellular pathogen load analysis

MH-S cells adhered for 4 hours in T-25 flasks were infected with *M*. *smegmatis* at 10 multiplicity of infection (MOI) using 1x10^7^ bacteria to infect 1x10^6^ macrophages per ml culture. Three treatment sets (2 subsets each) were prepared: vehicle treatment before infection (“vehicle set”), 0.02% EO treatment before infection (“pre-treatment” set), 0.02% EO treatment after phagocytosis (“post-treatment” set). The macrophage-bacteria mixtures were incubated for 1 hour to allow phagocytosis to occur. The first subset of flasks from the ‘vehicle-treated set’ (control) and ‘pre-treatment set’ was analyzed for phagocytosis activity as follows. The cells were collected by centrifugation, made free of external bacteria using gentamycin (10 μg/ml), and subjected to cell lysis (using distilled water followed by SDS addition @ 0.25% and neutralization with albumin @ 0.1%). The lysate was agar plated to estimate the internalized bacterial number by colony forming units (CFU) analysis using the agar plating method. The second subset of flasks from the ‘vehicle-treated set’ (control), EO ‘pre-treatment set’, and EO ‘post-treatment set’ was allowed to further incubate for 24 hours and was subjected to internal CFU analysis (using the same assay as for phagocytosis) to assess the bacterial build up within macrophages in the presence and absence of EO. For 1,8-cineole experiments, the same treatment design as described above for EO was used. The lysate was serially diluted and plated on tryptic soy agar (TSA) for determination of the colony forming units (CFU).

### Analyses

#### Cell viability

Vehicle-treated and EO- or 1,8-cineole- treated cells were stained with 0.4% Trypan blue (Invitrogen, Carlsbad, CA, USA). The live cells (unstained) versus dead cells (blue-stained) were counted by hemocytometer using an inverted microscope. The average number of cells counted in the 4 gridded areas (16 large squares) of the hemocytometer were multiplied by 10^4^ and the dilution factor to obtain the cell count per ml.

#### Nitric oxide (NO)

NO in terms of nitrite was estimated from the culture supernatants using the commercial Griess reagent system as per manufacturer’s instructions (Promega, Madison, WI, USA).

#### Cytokines (ELISA)

Various cytokines were analyzed in supernatants from treated and untreated macrophage cultures from different treatment groups as described above using the cytokine-specific commercial ELISA kits as per the manufacturer’s protocol (Affymetrix Inc. eBioscience San Diego, CA, USA); intracellular levels of IL-α and IL-β were also analyzed using cell lysates. Concentrations of the cytokines were estimated from the standard curves that were generated using known concentrations of the corresponding purified recombinant cytokines provided in the respective ELISA kits.

#### Western blot analysis

Various signaling targets (MAPKs, NFκB, NLRP3) were analyzed using total protein extracts prepared from control (vehicle only) and treated [(EO alone or LPS alone or EO+LPS) or (cineole alone or cineole +LPS)] cells. The cells were lysed (coinciding with 30 minutes or 4 hours post-LPS addition as described above) using radio immunoprecipitation assay (RIPA) buffer (20 mM tris.HCl pH 7.5, 1 mM EDTA, 1% nonidet P-40, 0.1% sodium deoxycholate, 2.5mM sodium pyrophosphate, 150 mM NaCl) supplemented with protease and phosphatase inhibitors. Protein extracts from different treatments were resolved on a 12% SDS-PAGE gel and analyzed by Western blot analysis using total- and phospho- antibodies for MAPKs (p38, JNK, ERK) and NFκB and antibodies for NLRP3 using ECL kit (Pierce Chemical, Rockford, IL, USA). Primary rabbit antibodies (Cell Signaling Technology, Danvers, MA, USA) were used at a dilution of 1:1000 for all test targets, including MAPKs (p38 or p-p38, SAPK/JNK or p-SAPK/JNK, p44/42/ERK/1/2), NFκB, and NLRP3, whereas the β- Actin antibody (Sigma, USA) was used at a 1:4000 dilution. HRP-conjugated anti-rabbit IgG secondary antibody (Sigma, USA) was used at a 1:4000 dilution. Bands were visualized using ECL kit (Pierce Chemical, Rockford, IL, USA) and quantified using NIH Image J software.

#### Quantitative RT-PCR analysis

Total RNA from cells, pre-treated with either EO or 1,8-cineole for 3 hours followed by a 30 minute LPS challenge, was isolated using Tri-reagent according to the manufacturer’s protocol (MRC Inc., Cincinnati, OH, USA). Quantitative RT-PCR was done using Brilliant III Ultra-Fast SYBR^®^ Green QRT-PCR master mix per the manufacturer’s protocol (Agilent Technologies, USA) using gene-specific primers listed in Table 1. The housekeeping gene *GAPDH* was used as internal control for normalizations. Impact of EO or cineole pre-treatment on expression of target gene transcript was presented in terms of fold-change in mRNA levels, calculated by comparing with the untreated and internal controls, per the following formula: (2^-ΔΔCT^), where ΔΔCT is the difference between ΔCT value of experimental and ΔCT value of controls for fold change as described in our previous report [19].

#### Statistical analysis

The data represent means ± standard error of the means (SEM) obtained from three independent treatments. Statistical analysis was performed using one-way ANOVA and differences between multiple groups were determined by the Bonferroni- Holm post hoc test. P-value ≤ 0.05 was accepted as statistically significant.

## Results

### Dose optimization for eucalyptus oil (EO) and 1,8-cineole

**Fig 1:**
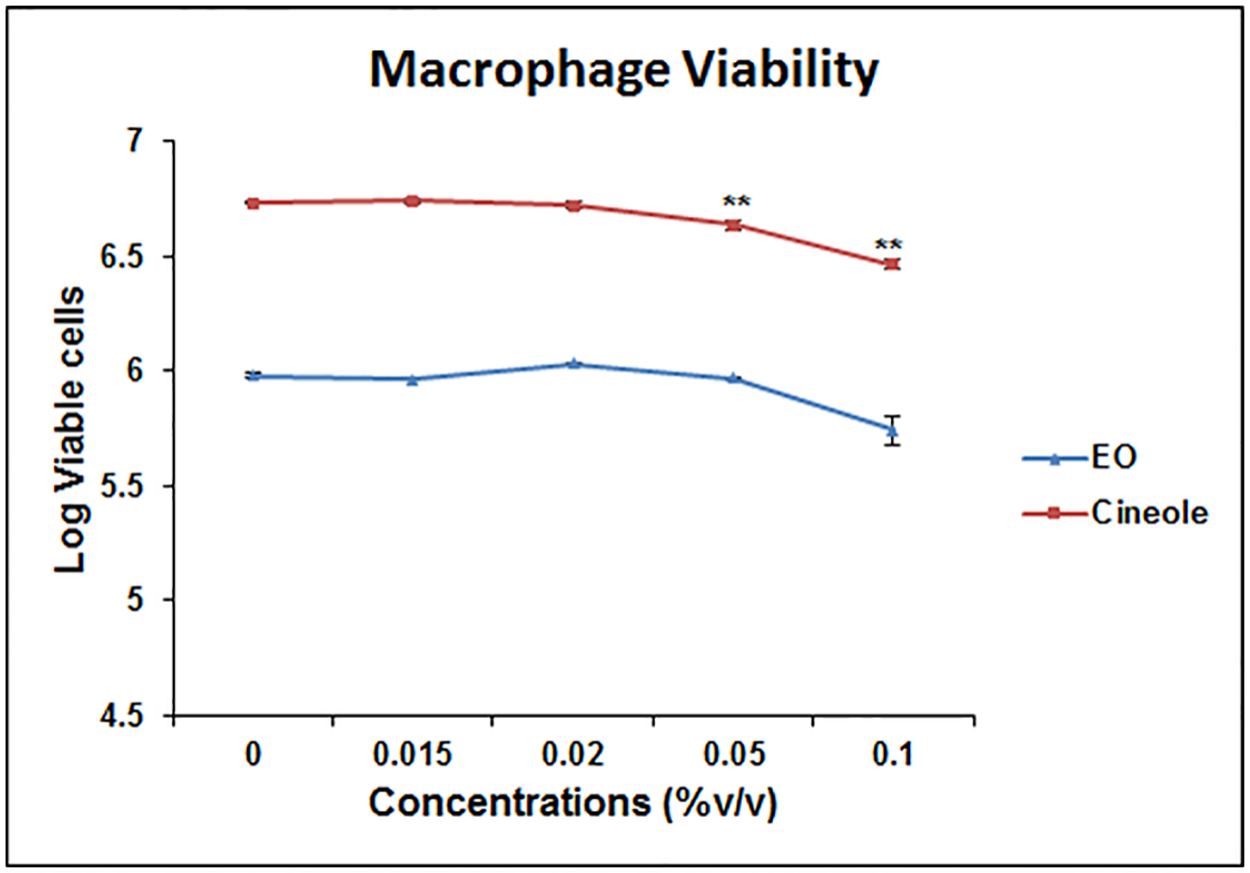
Selection of non-cytotoxic concentration of eucalyptus oil (EO) for treatment of alveolar macrophages. Murine alveolar macrophage cell line MH-S (1×10^6^ cells/well) was cultured in presence of varying concentrations of EO (0.01–0.10%) for 24 hours and cell viability (%) was assessed using Trypan blue staining. Values represent mean ± SEM based on three independent experiments. Asterisks (**) indicate statistically significant (*P* ≤ 0.01, respectively) difference when compared to the vehicle control.

To determine a noncytotoxic concentration of eucalyptus oil (EO) and its constituent 1,8-cineole, the MH-S cells were treated with increasing amounts of EO or 1,8-cineole and were compared for cell viability with the vehicle-treated group. The results showed that EO and 1,8 cineole do not adversely affect the viability of lung macrophages (Fig 1) up to a reasonably high concentration (0.05%); the 0.02% concentration appeared to be the safe level to use and was therefore employed in all subsequent experiments.

### Inhibition of pro-inflammatory response by EO and its constituent

**Fig 2:**
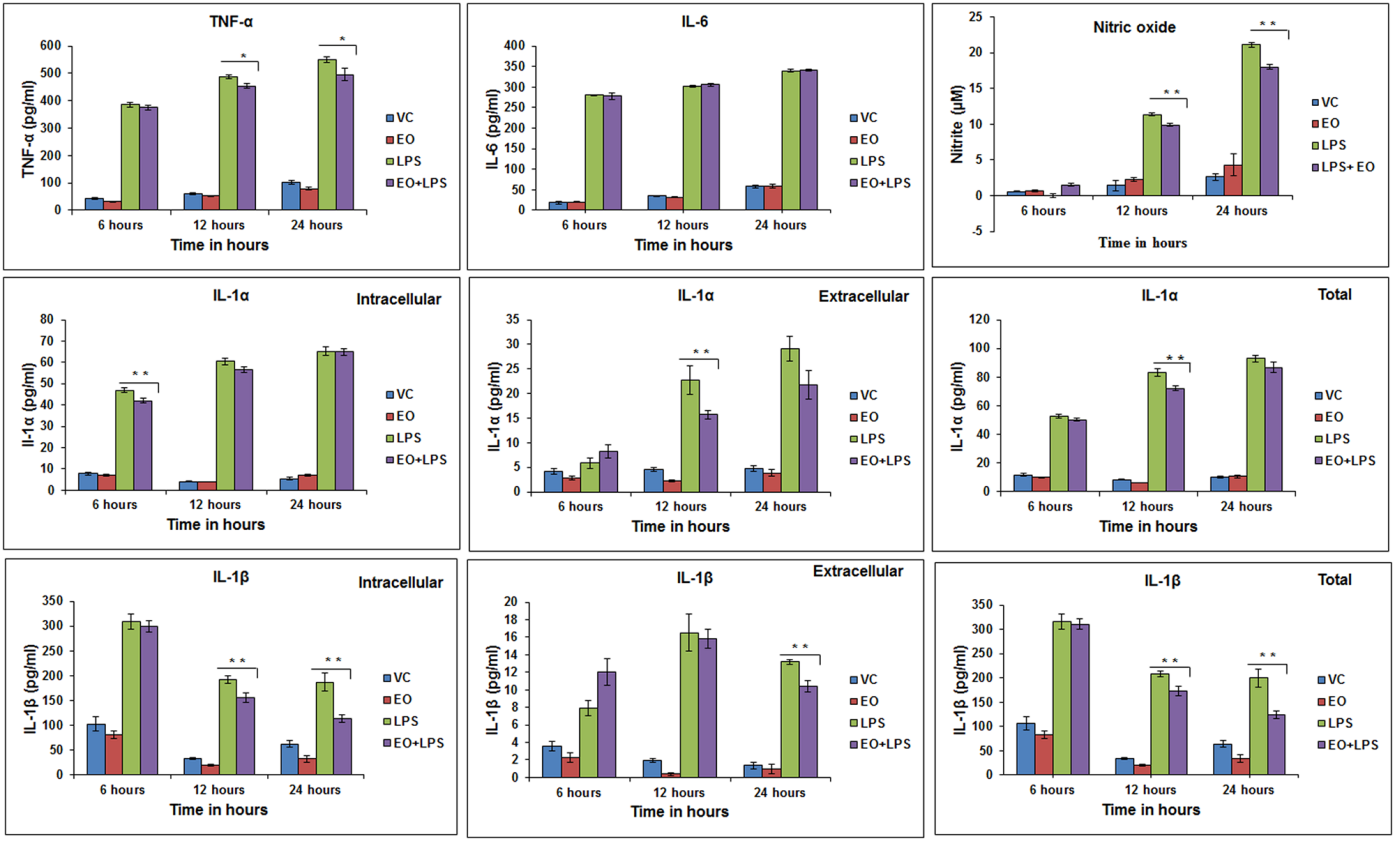
Modulation of LPS-induced pro-inflammatory response in alveolar macrophage cells by pre-treatment with eucalyptus oil (EO). MH-S cells (1x10^6^ cells/ml culture/well) were treated with (i) Vehicle (0.1% MeOH), (ii) EO only (0.02% vol/vol), (iii) LPS only (2 μg/ml), and (iv) LPS (2 μg/ml) +EO (0.02%); EO was added 3hours before LPS addition (“pre–treatment”). NO and cytokines were periodically measured in the culture supernatant through 24 hour time-point. Values are presented as mean ± SEM based on three independent treatments. Asterisks (* and **) indicate statistically significant (*P* ≤ 0.05 and *P* ≤ 0.01, respectively) difference when compared to the positive control (LPS only).

**Fig 3:**
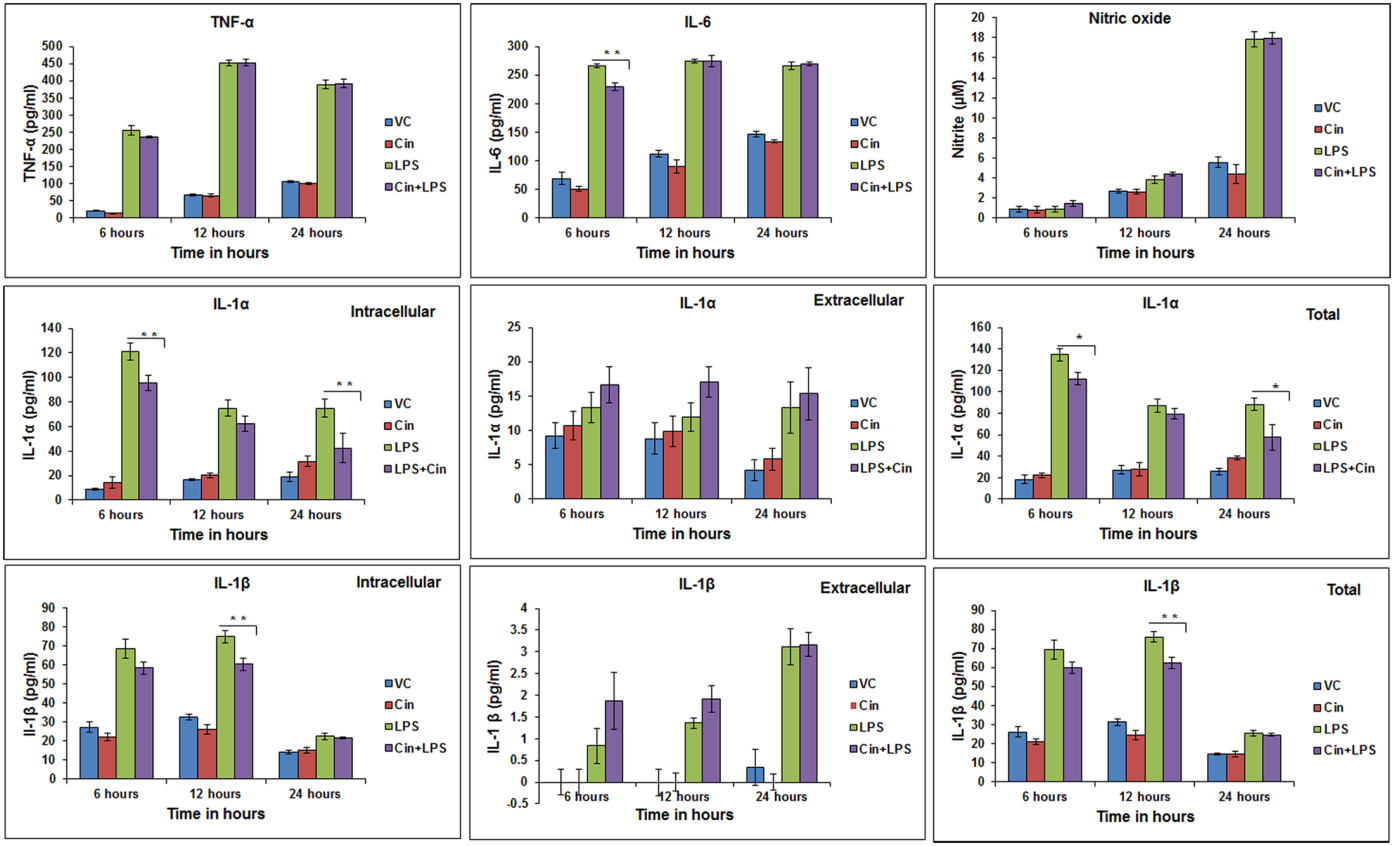
Modulation of LPS-induced pro-inflammatory response in alveolar macrophage cells by pre-treatment with 1,8-cineole (Cin). MH-S cells (1x10^6^ cells/ml culture/well) were treated with i) vehicle (0.1% MeOH), (ii) Cin only (0.02% vol/vol), (iii) LPS only (2 μg/ml), (iv) LPS (2 μg/ml) +Cin (0.02%); Cin was added 3hours before LPS addition (“pre-treatment”). NO and cytokines were periodically measured in the culture supernatant up to 24 hour time-point. Values are presented as mean ± SEM of based on three independent treatments. Asterisks (*) and (**) indicate statistically significant (*P* ≤ 0.05 and *P* ≤ 0.01, respectively) difference as compared to the positive control (LPS only).

The selected test concentration (0.02%) of EO (or its constituent 1,8-cineole) by itself caused no induction of inflammatory response when compared to the vehicle-only control (Figs 2 and 3). LPS-treatment alone induced a significant pro-inflammatory response in cultured AMs in terms of pro-inflammatory cytokines (TNF-α, IL-1α, IL-1β and IL-6) and NO, unlike the vehicle-only and EO-only treated groups. The individual inflammatory mediators increased with time of incubation in the 24 hour treatment regimen. This pro-inflammatory response was significantly (P ≤ 0.01 or P ≤ 0.05) reduced when the cells were pre-treated with EO (0.02%) for 3 hours before the LPS challenge, although the effect varied with the inflammatory mediator and the time-point of incubation (Fig 2). IL-6 was an exception which showed no modulation by EO pre-treatment. TNF-α levels were impacted at 12 and 24 hours (P ≤ 0.05) in case of pre-treatment with EO (Fig 2). Intracellular and extracellular levels of IL-1α were significantly impacted at 6 and 12 hour time-points (P ≤ 0.01), respectively and a significant reduction in total level (intracellular + extracellular) of IL-1α was observed at 12 hours (P ≤ 0.01). Intracellular levels of IL-1β (pro IL-1β form) were significantly impacted at 12 and 24 hours (P ≤ 0.01) (Fig 2) while the extracellular levels of IL-1β were significantly impacted at 24 hours ((P ≤ 0.01). Total level of IL-1β was significantly reduced at 12 and 24 hours (P ≤ 0.01). Pre-treatment with EO significantly reduced the LPS-induction of NO at 12 and 24 hour time-points (P ≤ 0.01 (Fig 2).

When compared to EO, 1,8-cineole pre-treatment showed an opposite or divergent effect on certain inflammatory mediators such as a reduction in IL-6 level (6 hours) and no effect on TNF-α and NO (Fig 3). 1,8-cineole pre-treatment significantly reduced (P ≤ 0.01) intracellular levels of IL-1α at 6 and 24 hour time points and IL-1β at 12 hours. No significant reduction in extracellular levels of IL-1α and IL-1β was observed (Fig 3). The overall level of IL-1α therefore showed a significant decrease at 6 and 24 hours (P ≤ 0.05) and that of IL-1β at 12 hours (P ≤ 0.01).

### Modulation of phagocytosis activity and intracellular pathogen load in a mycobacterial infection model

**Fig 4:**
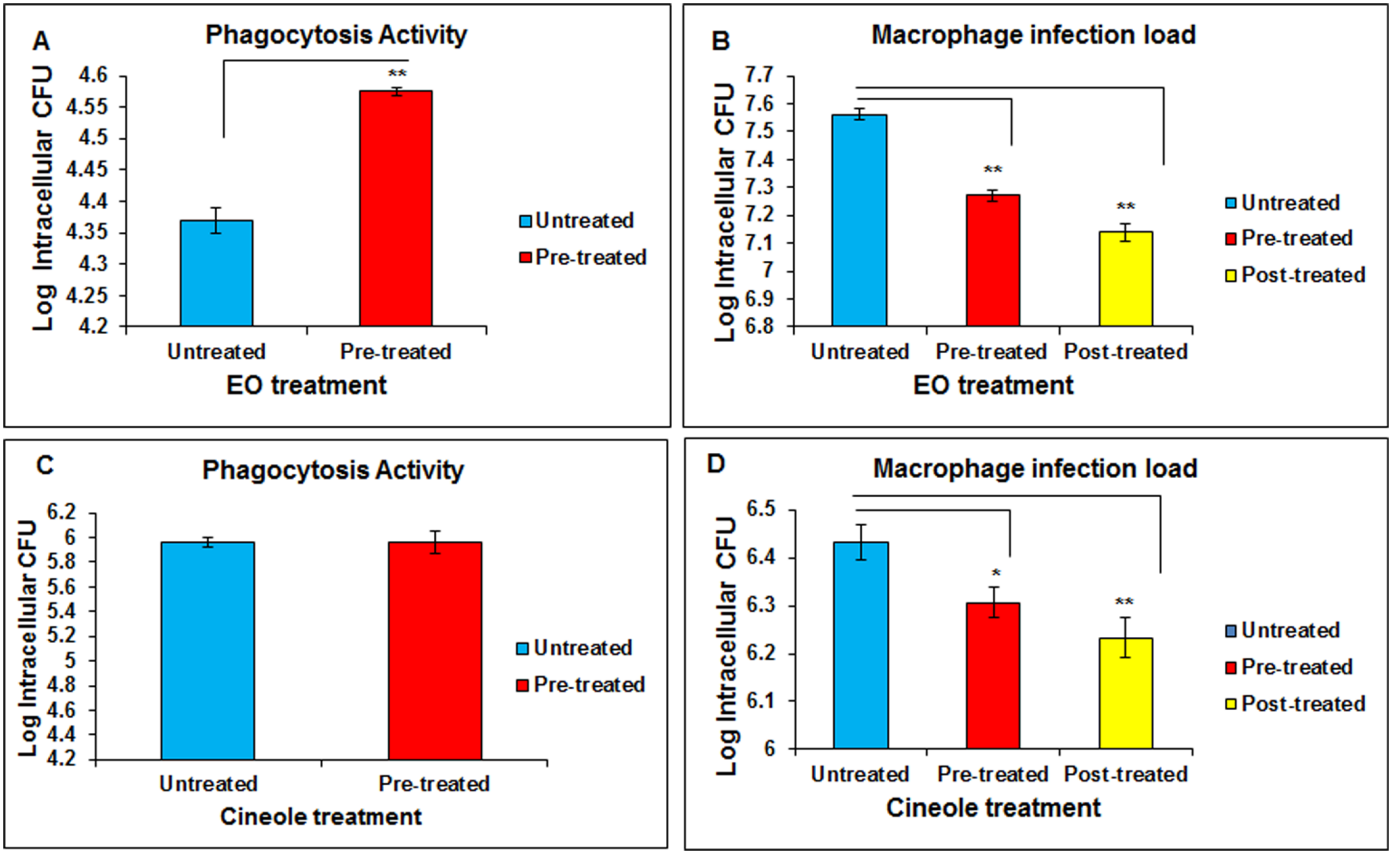
Effect of eucalyptus oil (EO) and 1,8-cineole on phagocytosis of *Mycobacterium smegmatis* and its clearance during infection of alveolar macrophages. Panels A and C: Effect of EO and cineole on phagocytosis activity (1h); Panels B and D: Effect of EO and cineole on bacterial clearance (24 h). MH-S cells (1x10^6 cells/ml culture/well) were treated with EO (0.02% v/v) or cineole either 3h before phagocytosis (“pre-treatment”) or right after phagocytosis (“post-treatment”) during infection with *M*. *smegmatis*. Bacterial counting (CFU analysis) was performed on macrophage cell lysates at 1hour (phagocytosis) or 24 hours (pathogen load) post-infection challenge. Values are presented as mean ± SEM based on three independent treatments. Asterisks (*) and (**) indicate statistically significant (*P* ≤ 0.05 and P ≤ 0.01, respectively) difference as compared to the vehicle control.

In the murine AM infection model meant to mimic mycobacterial infections in alveolar macrophages, EO pre-treatment caused significant enhancement in the phagocytosis activity (Fig 4A) and a considerable increase in bacterial clearance (P ≤0.01) (reduction in intracellular bacterial build-up) of this mycobacterial species (Fig 4B). For 1,8-cineole, though pre-treatment did not enhance the phagocytic activity significantly as compared to the untreated group (Fig 4C), there was a significant increase in bacterial clearance in both pre-treatment (P ≤0.05) and post-treatment (P ≤0.01) groups (Fig 4D). This coincided with a parallel slight increase, albeit non-significant (P >0.05), in the anti-mycobacterial cytokine TNF-α levels in the EO-pretreated macrophages (data not shown). Post-phagocytosis treatment with EO showed a relatively greater response as compared to the pre-treatment.

### Modulation of pro-inflammatory innate immune signaling by EO and 1,8-cineole

#### Phosphoactivation of MAPKs, NFκB, and NLRP3

**Fig 5:**
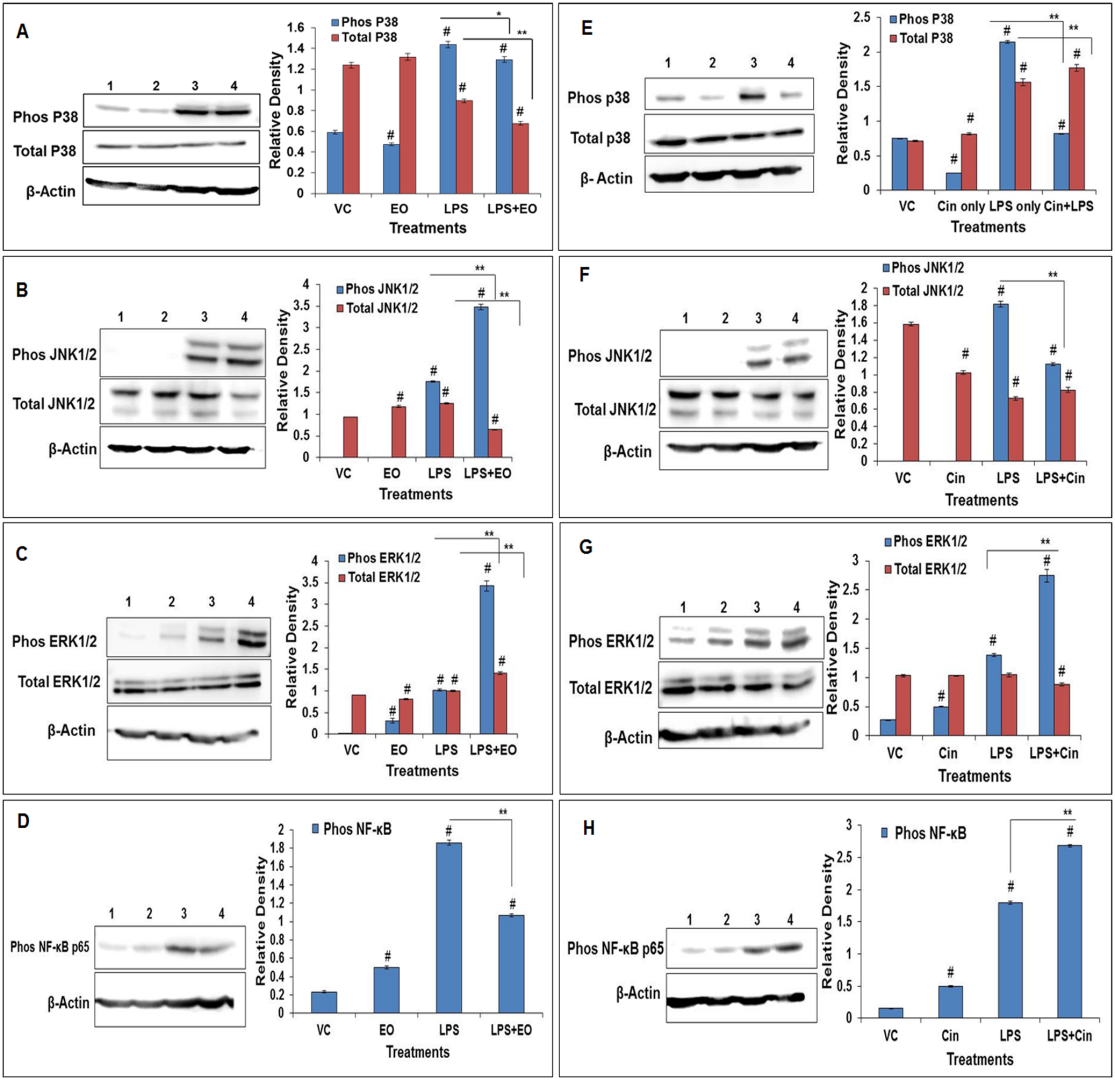
Modulation of LPS-activation of MAPKs in alveolar macrophages by pre-treatment with eucalyptus oil (EO) or 1,8-cineole (Cin). (A-D) EO pre-treatment blots; (E-H) Cin pre-treatment blots for p38, SAPK/ JNK, ERK1/2, and NF-kB. MAPKs, respectively. Activation was assessed in terms of increase in both the total content and the phosphorylated form; NF-kB activation was assessed in terms of increase in its phosphorylated form. Densitometry analysis of Western blots was done using NIH software image J. Lanes1-4 represent vehicle control (VC), EO-only, LPS-only and EO+LPS for EO pre-treatment group or VC, Cin, LPS and Cin +LPS for Cin pre-treatment group, respectively. Details on the treatments and antibodies for total- and phospho- MAPKs and β-Actin are described in Materials and Methods section. Values represent means ± SEM based on three independent treatments. Asterisks (*) and (**) indicate statistically significant (*P* ≤ 0.05 and *P* ≤ 0.01, respectively) difference as compared to the LPS treatments while the number sign (#) indicates statistical significance as compared to the vehicle control. See S1 File for the original Western blot images.

As expected, LPS significantly induced the phosphorylation of MAPKs which are known to activate the downstream signaling cascade including transcription factors and effector molecules (soluble mediators) during LPS-induced inflammatory response. In the EO or 1,8-cineole pre-treated group, there was a significant increase in phosphorylated ERKs (ERK1/2) and a significant reduction in phosphorylated p38. The phosphorylated forms of JNK1/2 were increased by EO and decreased by 1,8-cineole, while phospho-NFκB (P65) was decreased by EO and increased by 1,8-cineole (Fig 5). In terms of total content, ERK1/2 levels were also significantly increased while total p38 and JNK1/2 kinase levels were reduced in the EO pretreated group. On the other hand, pre-treatment with 1,8-cineole led to an increase in total p38 but no change in total ERK1/2 and JNK1/2 levels when compared with the LPS-only treated group (Fig 5).

**Fig 6:**
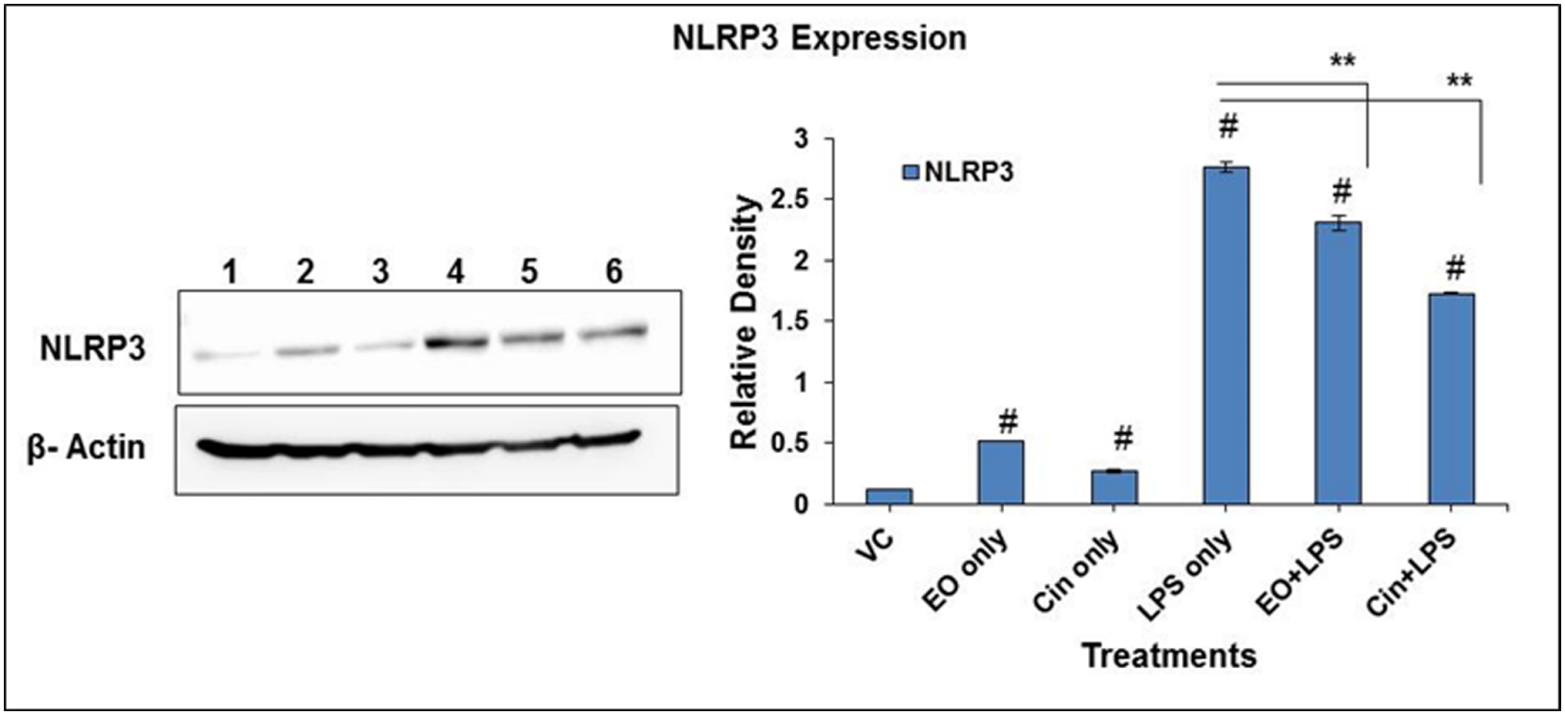
Modulation of LPS-activation of NLRP3 inflammasome in alveolar macrophages by pre-treatment with eucalyptus oil (EO) or 1,8-cineole (Cin). NLRP3 protein expression was assessed in cell lysates of EO- and Cin- pretreatment groups (3 hours pre-treatment with either EO or Cin at 0.02% concentration followed by induction with 2 μg/ml LPS for 4 hours). Cell lysates were analyzed by Western blotting using anti-NLRP3 antibodies. Densitometry analysis of Western blots was done using NIH software image J. Lanes1-6 represent VC, EO, Cin, LPS, and EO+LPS, Cin +LPS, respectively. Values represent means ± SEM based on three independent treatments. Asterisks (*) and (**) indicate statistically significant (*P* ≤ 0.05 and *P* ≤ 0.01, respectively) difference as compared to the LPS-only treatment while the number sign (#) indicates statistically significance as compared to the vehicle control. See S1 File for the original Western blot images.

LPS induces NLRP3 inflammasome activation which plays a vital role in IL-1 secretion and the inflammatory response. EO and 1,8-cineole pre-treatment significantly (P ≤ 0.01) reduced the LPS- induced NLRP3 activation, a critical step in activation of the inflammasome (Fig 6).

#### Transcriptional regulation of other key innate immune signaling targets

**Fig 7:**
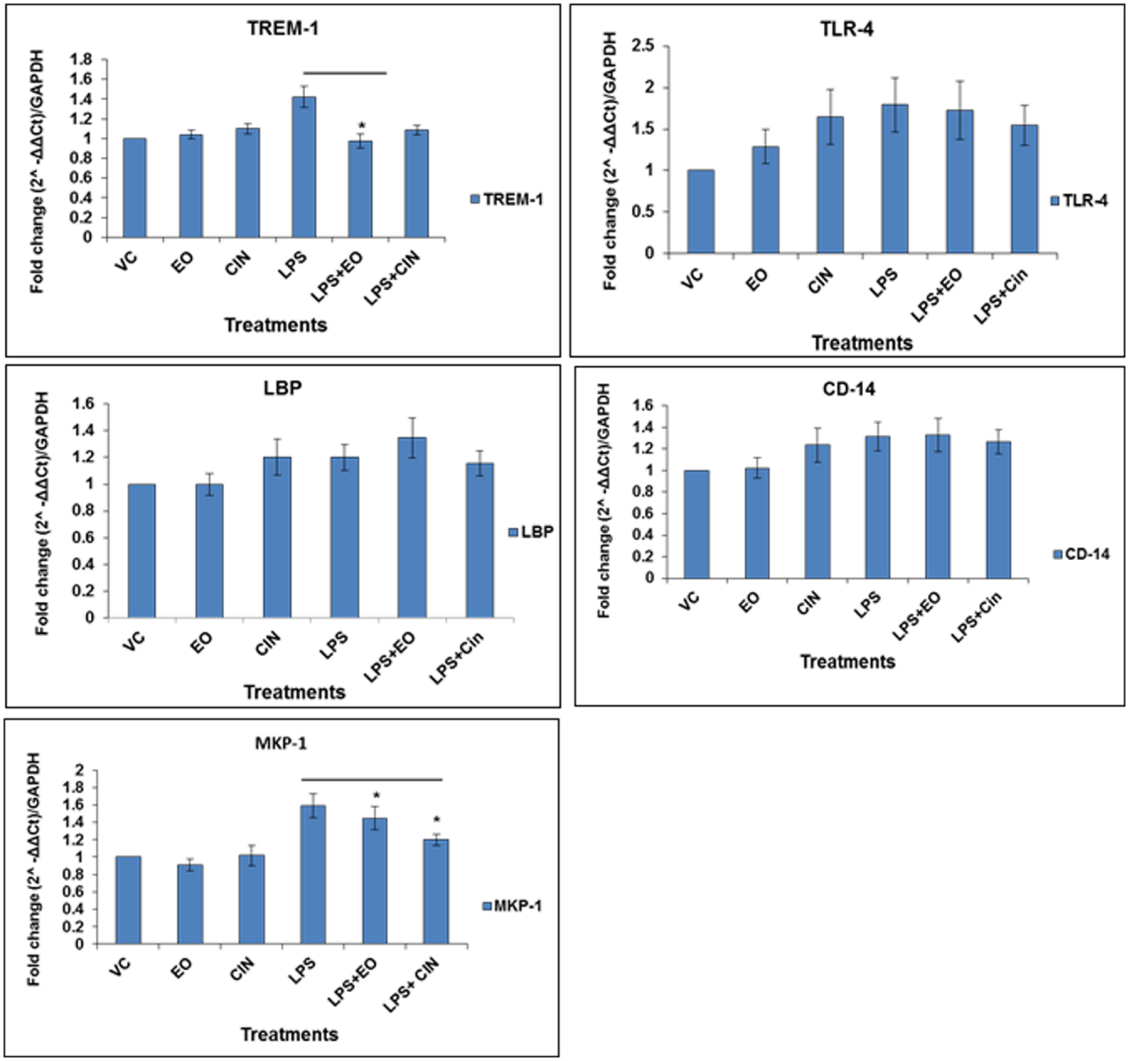
Modulation of mRNA expression of key target genes of LPS-signaling pathway by pre-treatment with eucalyptus oil (EO) and 1,8-cineole. **(A-E)** Target genes encoding MKP-1, TREM-1, LBP, TLR4 and CD14, respectively. mRNA expression was analyzed by quantitative RT-PCR using total RNA isolated from different treatment groups, as detailed in Fig 6 legend. The housekeeping gene GAPDH was used as internal control for normalizations and expression difference as fold-change was determined using the formula (2^-ΔΔCt^) method. Values are presented as mean ± SEM based on three independent treatment groups. Asterisks (*) and (**) indicate statistically significant (*P* ≤ 0.05 and *P* ≤ 0.01, respectively) difference as compared to the LPS-only treatment.

Gene expression of key targets of LPS signaling, including the surface receptors TLR4 (and its binding partners LBP and CD14) and TREM-1, and MAPKs phosphatase MKP-1, was monitored after 30 minutes of LPS treatment based on quantitative RT-PCR (Fig 7). Expression levels of TLR4, LBP and CD14 in the pretreated samples were not significantly different as compared to the LPS alone. In contrast, MKP-1 and TREM-1 mRNA levels in the EO or 1,8-cineole pre-treatment groups were significantly reduced as compared to the LPS-only group.

## Discussion

Despite a recognized role of EO in aroma therapy for lung conditions including inflammatory and infection ailments, there is a paucity of meaningful data on the mechanistic mode of action of this product in lung models (*in vitro* and *in vivo*). As EO originating from different sources/vendors may quantitatively vary in biological response because of variable proportions of the constituents, testing of individual constituents could provide vital mechanistic information. In this direction, using an established *in vitro* murine lung alveolar macrophage model, the MH-S cell line [20], and both EO and its major constituent 1,8-cineole, we observed inhibition of inflammatory and infection responses; the responses were measured in terms of pro-inflammatory mediators, phagocytosis and infection clearance, and activation of key receptors and transducers of the LPS–induced inflammation signaling pathway.

### Attenuation of inflammatory response

Inflammation is an important innate immune response triggered by external stimuli such as pathogens and pathogen products (e.g. LPS) or physical injury to combat the initial cause of threat and restore homeostasis. However, uncontrolled inflammation is deleterious to the tissues and can lead to various inflammatory conditions and cell/tissue damage [21]. While different immune cell types such as neutrophils, macrophages, eosinophils, lymphocytes may be impacted during inflammation [22–23], this study focused on macrophages which are known to play a key role in both infections and inflammation. Macrophages activated by inflammatory stimuli such as LPS in bacterial infections secrete pro-inflammatory cytokines such as TNF-α, IL-1, IL-6 and other soluble mediators such as nitric oxide (NO), which play a crucial role in induction and progression of the inflammatory response. Continuous activation of the macrophages may lead to elevated levels of these mediators which can lead to acute or chronic inflammatory conditions such as septic shock-like syndrome resulting in tissue damage and organ failure [21]. Hence, evaluation of novel candidates for their anti-inflammatory efficacy using this model inflammatory response paradigm in macrophages may help identify promising alternative or adjunct therapeutics.

In this study, pre-treatment of alveolar macrophages with EO before LPS challenge caused a reduction in pro-inflammatory soluble mediators, including cytokines such as TNF-α, IL-1β, IL-1α and NO (Fig 2). In comparison, 1,8-cineole also showed an anti-inflammatory effect, albeit for only certain mediators (intracellular IL-1α and IL-1β; IL-6 secretion), and the effects varied with the time-point of incubation (Fig 3). While this study showed significant attenuating effect at 0.02% level of either EO or 1,8-cineole, the extent may be concentration-dependent and increase at higher concentrations. Interestingly, anti-inflammatory effects of EO were also reported in human MDMs [24] and AMs isolated from COPD patients [25]. Taken together, we observed significant diminishing of potent pro-inflammatory mediators (cytokines and NO) by EO and 1,8-cineole in exposed lung macrophages, which explains the nature and scientific basis of their anti-inflammatory potential in aroma therapy.

### Enhanced pathogen clearance

The past decade has witnessed an unprecedented emergence of drug resistance strains of priority human pathogens such as *Mycobacterium tuberculosis* and *Staphylococcus aureus* (MRSA) [26–27]. Available synthetic drugs and antibiotics are proving less efficacious to meet the challenges posed by the drug resistant strains. Identification of naturally-derived compounds could provide alternate agents to combat the resistance problem because of their divergent mode of action. In this context, our results using a mycobacterial infection model are significant in that pre-treatment or post-treatment of macrophages with EO or 1,8-cineole significantly enhanced the intracellular pathogen clearance (Fig 4B and 4D). Similarly, pre-treatment with EO significantly enhanced the phagocytosis while 1,8-cineole pre-treatment had little though non-significant effect (Fig 4A and 4C). The EO enhancement of phagocytosis is consistent with that observed in human monocyte-derived macrophages (MDMs) [24]. A study on human neutrophils reported a decrease in phagocytosis activity by the Bay laurel (BL) and Cajuput (CA) extracts containing 1,8-cineole as the major component but contribution of other components in these extracts toward this inhibition cannot be ruled out [28]. Greater bacterial clearance in EO post-treatment might be because of an additive effect due to prolonged contact and possibly direct antimicrobial action of EO molecules, as demonstrated in our previous in vitro study [12]. Collectively, these results imply that EO and its constituents have the potential to be an alternate or adjunct anti-infective agent for combating mycobacterial infections of clinical importance.

### Orchestration of pro-inflammatory signaling pathways via transcriptional and post-translational modifications

In response to inflammation and infection stimuli, macrophages express different pattern recognition receptors (PRRs), including surface receptors such as toll-like receptors (TLRs), and trigger receptors expressed on myeloid cells 1 (TREM-1) and cytosolic receptors such as Nod-like receptors (NLRs), which may work in concert to activate common downstream innate signaling cascades comprised of MAP kinases and transcription factors [29]. Activation of MAP kinases (MAPKs) eventually leads to activation of the transcription factors NF-κB and AP-1[30–31] resulting in the expression of pro-inflammatory genes encoding various cytokines such as TNF-α, IL-1α, IL-6 and enzymes catalyzing the generation of reactive oxygen species or reactive nitrogen species (e.g. NO) in the activated macrophages in response to external stimuli.

We investigated the effect of EO and 1,8-cineole on surface PRRs of the LPS-induced inflammation signaling pathway. Transcriptional (mRNA expression) analysis of TLR4 receptor and its partners (LBP and CD14) in the LPS-induced TLR4 receptor signaling pathway did not show any effect of pre-treatment either with EO or 1,8-cineole, suggesting that immune-modulation by these natural products during LPS-induced inflammation might be restricted to targets downstream of the TLR4 receptor signaling complex. Another surface receptor, TREM-1, has been known to be upregulated in septic shock-like conditions involving LPS and is highly expressed in monocytes in bacteria-infected human tissues [32–33]. TREM-1 expression has been shown to amplify the TLR-mediated inflammation pathway [32–33]. In our study, EO pre-treatment significantly decreased LPS-induced mRNA expression of TREM-1 (Fig 7). 1,8-cineole pre-treatment also reduced the expression, but the difference was not statistically significant. The observed reduction in TREM-1 expression by EO is a significant finding as it opens up novel avenues for expanded studies on attenuation of inflammation by EO and its components, especially in inflammatory health conditions, where the role of the TREM-1 pathway is critical.

Activation of Nod-like receptors (NLRs), the cytosolic pattern recognition receptors (PRRs), in macrophages is critical for the innate immune response to inflammation- and infection- stimuli. Unlike TLRs, which recognize pathogen associated molecular patterns (PAMPs) on the surfaces, NLRs are activated inside the cell and form a part of the inflammasome [34]. Inflammasomes are formed by the multiprotein complexes with different NLRs such as NLRP1b, NLRP3 and NLRC4. Active inflammasomes can lead to the caspase 1-mediated activation of the highly inflammatory cytokines IL-1β and IL18 [35–36]. Therefore, regulation of this potent inflammatory signaling complex is critical in inflammatory conditions. Pre-treatment either with EO or its component 1,8-cineole significantly attenuated IL-1β levels, which coincided with downregulation of the NLRP3 receptor of the inflammasome complex (Fig 6). This observation further emphasizes the potent anti-inflammatory activity of EO and its component as an alternate therapy for various inflammatory diseases involving the inflammasome. Further studies in this direction could elucidate information regarding the modulation of other components of the inflammasome.

In pro-inflammatory signaling pathways, MAPKs (p38, JNK1/2, ERK1/2) and NF-κB are activated by phosphorylation at threonine or tyrosine residues of the inactive forms (non-phosphorylated), conveying the signal from the cell membranes to the nucleus and thus controlling the expression of different inflammatory mediators [21]. Further, the MAPKs can in turn phosphorylate other protein kinases known as MAPK-activated protein kinases (MAPKAPK) [21]. Alternately, MAPKs may be dephosphorylated by MAP kinase phosphatase MKP-1[37–38] to maintain immune homeostasis. When pretreated with EO, the LPS-induced phosphorylation of key signal transducers NFκB and p38 was decreased while that of the other MAPKs (ERK1/2 and JNK1/2) was increased (Fig 5). Increase in ERK1/2 phosphorylation also points to the anti-inflammatory effect of EO/Cineole, as demonstrated for a plant derived flavonoid wogonin [39]. The attenuation in phosphorylation of NF-κB and p38 was in agreement with the decrease in key inflammatory mediators, including cytokines (TNF-α, IL-1α and IL-1β) and NO. Although 1,8-cineole treatment did attenuate the phosphorylation of p38 (and JNK1/2) and increased ERK1/2 phosphorylation (Fig 5), it did not show as much of an attenuating effect as EO on the production of inflammatory mediators. One reason for this divergence may be that 1,8-cineole increased phospho-NFκB unlike EO and this might have reversed in part the attenuating effect of MAPK modulation. Taken together, EO and 1,8-cineole might be acting at different targets to differentially modulate the phosphoactivation of MAPKs and NFκB, thereby divergently controlling the LPS-mediated inflammatory responses.

NO production is caused by overexpression of inducible nitric oxide synthase (iNOS) [40] which is particularly induced via the NF-κB pathway [41] either directly or by inflammatory cytokines. For instance, inhibition of TNF-α converting enzyme (TACE) by gene silencing reduced the local inflammation in a rabbit animal model via reduced iNOS expression [42]; use of iNOS inhibitor reduced pulmonary inflammation in mouse models [43]. In this context, it is therefore significant that EO pre-treatment caused a reduction in LPS-induced NO production in our model, which coincided with a decrease in the activated form of NF-κB (Figs 2 and 3). In contrast, 1,8-cineole pre-treatment did not reduce either the NO levels or the phosphorylated form of NF-κB (which was elevated instead), thus suggesting a divergent mode of regulation by 1,8-cineole.

To combat the deleterious effect of stimulation of the PRRs and MAPKs, the phosphatase MKP-1 is induced during LPS signaling as a key negative regulator of MAPKs phosphorylation. MKP-1 generally dephosphorylates MAPKs, thus attenuating the pro-inflammatory cytokine response [44] by negatively regulating the MAPK activities [45]. On the other hand, absence of this protein such as in DUSP1-deficient (DUSP1-/-) mice, has been shown to increase the activation of p38 and cytokine production in bone marrow-derived macrophages. Further, DUSP1-/- mice showed increased lethality and overproduction of TNF-α and IL-6 upon LPS challenge [46]. While the activity of MKP-1 protein is known to be post-translationally regulated (phosphorylation and acetylation) to help interact with MAPKs [44], initial level of induction of the MKP-1 by LPS may be critical. Pretreatment with EO or 1,8-cineole reduced the MKP-1 mRNA levels (Fig 7), suggesting a transcriptional downregulation of MKP-1; this in turn might have led to elevated levels of phosphorylated ERK1/2, thereby favoring a diminished inflammatory response.

## Conclusions

**Fig 8:**
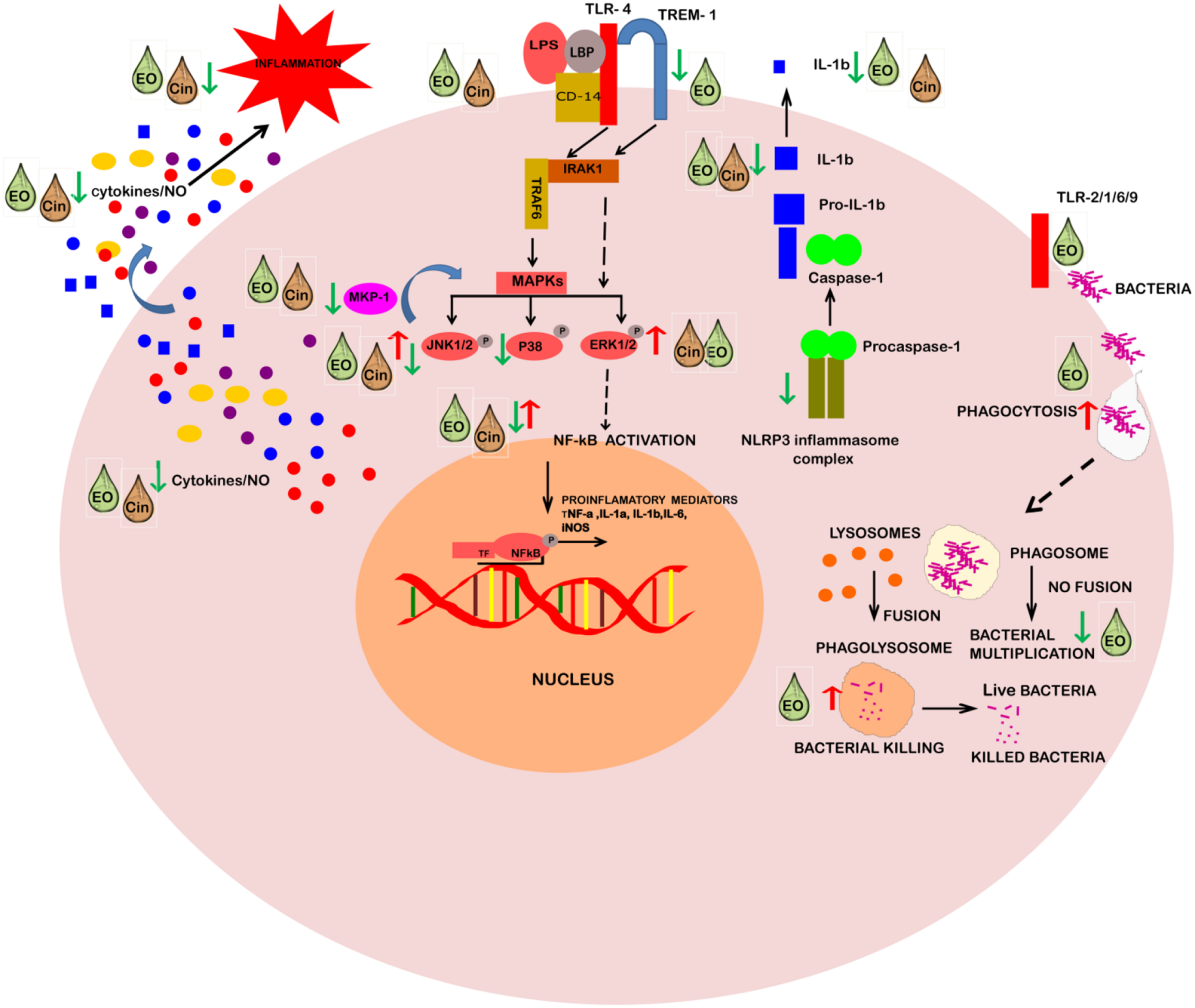
Schematic representation of immune-modulatory mode of action of eucalyptus oil and its constituent 1, 8-cineole on different targets of LPS/infection-induced pathways in alveolar macrophage. *Abbreviations*: EO, Eucalyptus oil; Cin, 1,8-Cineole; LPS, Lipopolysaccharide; TLR, Toll-like receptor; LBP, LPS-binding protein; CD14, Cluster of differentiation 14; TREM-1, Triggering receptor expressed on myeloid cells 1; IRAKs, IL-1 Receptor-Associated Kinases; TRAF6, TNF receptor-associated factor 6; MAPKs, Mitogen-activated protein kinases; ERK, Extracellular signal–regulated kinases; JNK: c-Jun N-terminal kinases; MKP-1, MAP kinase phosphatase-1; NF-κB, Nuclear factor kappa-light-chain-enhancer of activated B cells; NLRP3, Nod-like receptor family pyrin domain containing 3; NO, Nitric oxide: TF, Transcription factor.

Taken together, this study demonstrates the nature and mechanistic basis of the potent anti-inflammatory and anti-infective properties of EO and its constituent 1,8-cineole in lung inflammation- and infection- models based on alveolar macrophages. Future studies on human macrophages and other immune cells may yield more comprehensive information on these properties. In terms of in vivo anti-infective activity, EO stimulated phagocytosis and pathogen clearance of mycobacteria in lung macrophages. Similarly, 1,8-cineole was also effective in mycobacteria clearance in infected AMs. For anti-inflammatory activity, while EO seemed more versatile than its constituent 1,8-cineole, both significantly attenuated IL-1 cytokines. These immunomodulatory effects coincided with alterations in both upstream and downstream signaling arms of the LPS-induced inflammatory signaling pathway (Fig 8). Particularly, the attenuation of the IL-1 response by both EO and 1,8-cineole coincided with their common ability to diminish activation of the NLRP3 inflammasome. Downregulation of surface pattern recognition receptor TREM-1 by EO was in alignment with modulation of the downstream activation of the signaling cascade (MAPKs and NF-κB) and diminishing of the overall proinflammatory response to LPS. Furthermore, the MAP kinase phosphatase MKP-1, a key negative regulator of MAPKs, was also altered by EO and 1,8-cineole. Collectively, the identified key upstream receptor targets and specific downstream signaling events impacted by EO and its constituent 1,8-cineole could provide novel avenues for future development of EO-derived alternative or adjunct therapeutic leads against inflammatory or infection conditions of clinical importance.

## Supporting information

S1 File Western blot images for Figs 5 and 6. For Fig 5: (A-D) EO pre-treatment blots; (E-H) Cin pre-treatment blots for p38, SAPK/ JNK, ERK1/2, and NF-kB. MAPKs, respectively. Lane M represent protein molecular weight ladder, lanes1-4 represent vehicle control (VC), EO-only, LPS-only and EO+LPS for EO pre-treatment group and VC, Cin, LPS and Cin +LPS for Cin pre-treatment group, respectively. For Fig 6: Lane M represent protein molecular weight ladder, lanes1-6 represent VC, EO, Cin, LPS, and EO+LPS, Cin +LPS, respectively.
(PDF)

## Abbreviations

CFU: Colony forming units
EO: Eucalyptus oil
ERK: Extracellular signal–regulated kinases
JNK: c-Jun N-terminal kinases
LPS: Lipopolysaccharide
MAPKs: Mitogen-Activated Protein Kinases
MOI: Multiplicity of Infection
NO: Nitric oxide
p38: the p38 mitogen-activated protein kinase

## References

[1] Sarkar S, Mazumder S, Saha SJ, Bandyopadhyay U. Management of inflammation by natural polyphenols: A comprehensive mechanistic update. Curr Med Chem. 2016; 23(16): 1657–1695. 27087243 10.2174/0929867323666160418115540

[2] Tabutia JRS, Kukundab CB, Waako PJ. Medicinal plants used by traditional medicine practitioners in the treatment of tuberculosis and related ailments in Uganda. J Ethnopharmacol. 2010; 127: 130–136. doi: 10.1016/j.jep.2009.09.035 19799983 10.1016/j.jep.2009.09.035

[3] Tsai ML, Lin CC, Lin WC, Yang CH. Antimicrobial, antioxidant, and anti-inflammatory activities of essential oils from five selected herbs. Biosci Biotechnol Biochem. 2011; 75(10): 1977–1983. 21979069 10.1271/bbb.110377

[4] Laird K, Phillips C. Vapor phase: a potential future use for essential oils as antimicrobials. Letters in Appl Microbiol. 2012; 54(3): 169–174. 10.1111/j.1472-765X.2011.03190.x 22133088

[5] Vuong QV, Chalmers AC, Jyoti Bhuyan D, Bowyer MC, Scarlett CJ. Botanical, phytochemical, and anticancer properties of the Eucalyptus species. Chem Biodivers. 2015; 12(6): 907–924 doi: 10.1002/cbdv.201400327 26080737 10.1002/cbdv.201400327

[6] Kumar HD, Laxmidhar S. A review on phytochemical and pharmacological of *Eucalyptus globulus*: a multipurpose tree. Int J Res Ayurveda Pharm. 2011; 2(5): 1527–1530.

[7] Jun YS, Kang P, Min SS, Lee JM, Kim HK, Seol GH. Effect of eucalyptus oil inhalation on pain and inflammatory responses after total knee replacement: A randomized clinical trial. Evid Based complement alternat Med. 2013; 2013: 502727 doi: 10.1155/2013/502727 23853660 10.1155/2013/502727 PMC3703330

[8] Silva J, Abebe W, Sousa SM, Duarte VG, Machado MIL, Matos FJA. Analgesic and anti-inflammatory effects of essential oils of Eucalyptus. J Ethnopharmacol. 2003; 89(2–3): 277–283. 14611892 10.1016/j.jep.2003.09.007

[9] Santos FA, Rao VS. Anti- inflammatory and antinociceptive effects of 1,8-cineole a terpenoid oxide present in many plant essential oils. Phytother Res. 2000; 14(4): 240–244. 10861965 10.1002/1099-1573(200006)14:4<240::aid-ptr573>3.0.co;2-x

[10] Juergens UR, Engelen T, Rack´e K, St¨ober M, Gillissen A, Vetter H. Inhibitory activity of 1,8-cineol (eucalyptol) on cytokine production in cultured human lymphocytes and monocytes. Pulm Pharmacol Ther. 2004; 17(5): 281–287. doi: 10.1016/j.pupt.2004.06.002 15477123 10.1016/j.pupt.2004.06.002

[11] Angela E, Sadlon ND, Lamson DW. Immune-modifying and antimicrobial effects of Eucalyptus oil and simple inhalation devices. Altern Med Rev. 2010; 15(1):33–47. 20359267

[12] Yadav N, Yadav E, Yadav JS. Antimicrobial activity of selected natural products against Gram-positive, Gram-negative and Acid-fast bacterial pathogens. Alternat Med Studies. 2012; 2(3): e13.

[13] Cermelli C, Fabio A, Fabio G, Quaglio P. Effect of Eucalyptus essential oil on respiratory bacteria and viruses. Curr Microbiol. 2008; 56(1): 89–92. doi: 10.1007/s00284-007-9045-0 17972131 10.1007/s00284-007-9045-0

[14] Ramos Alvarenga RF, Wan B, Inui T, Franzblau SG, Pauli GF, Jaki BU. Airborne antituberculosis activity of *Eucalyptus citriodora* essential oil. J Nat Prod. 2014; 77(3): 603–610. doi: 10.1021/np400872m 24641242 10.1021/np400872m

[15] Mulyaningsih S, Sporer F, Reichling J, Wink M. Antibacterial activity of essential oils from Eucalyptus and of selected components against multidrug-resistant bacterial pathogens. Pharm Biol. 2011; 49(9): 893–899. doi: 10.3109/13880209.2011.553625 21591991 10.3109/13880209.2011.553625

[16] Camporese A. In vitro activity of *Eucalyptus smithii* and *Juniperus communis* essential oils against bacterial biofilms and efficacy perspectives of complementary inhalation therapy in chronic and recurrent upper respiratory tract infections. Infez Med. 2013; 21(2): 117–124. 23774975

[17] Chandra H, Yadav E, Yadav JS. Alveolar macrophage response to the hypersensitivity pneumonitis pathogen *Mycobacterium immunogenum* is genotype-dependent and is mediated via JNK and p38 MAPK pathways. PLoS One. 2013; 8(12): e83172 doi: 10.1371/journal.pone.0083172 24349452 10.1371/journal.pone.0083172 PMC3859638

[18] Chandra H, Yadav JS. T-cell antigens of *Mycobacterium immunogenum*, an etiological agent of occupational hypersensitivity pneumonitis. Mol Immunol. 2016; 75: 168–177. doi: 10.1016/j.molimm.2016.05.020 27294559 10.1016/j.molimm.2016.05.020

[19] Frank EA, Carreira VS, Birch ME, Yadav JS. Carbon nanotube and asbestos exposures induce overlapping but distinct profiles of lung pathology in non-swiss albino CF-1 mice. Toxicol Pathol. 2016; 44(2): 211–225. doi: 10.1177/0192623315620587 26839332 10.1177/0192623315620587 PMC4976500

[20] Yadav N, Chandra H. Modulation of alveolar macrophage innate response in proinflammatory-, pro-oxidant-, and infection- models by mint extract and chemical constituents: Role of MAPKs. Immunobiol. 2017; https://doi.org/10.1016/j.imbio.2017.10.015. 10.1016/j.imbio.2017.10.015 29031422

[21] Moens U, Kostenko S, Sveinbjørnsson B. The role of Mitogen-Activated Protein Kinase-activated protein kinases (MAPKAPKs) in inflammation. Genes. 2013; 4: 101–133. doi: 10.3390/genes4020101 24705157 10.3390/genes4020101 PMC3899974

[22] Zhao C, Sun J, Fang C, Tang F. 1, 8-cineol attenuates LPS-induced acute pulmonary inflammation in mice. Inflammation. 2014; 37: 566–572. doi: 10.1007/s10753-013-9770-4 24197825 10.1007/s10753-013-9770-4

[23] Bastos VP, Gomes AS, Lima FJ, Brito TS, Soares PM, Pinho JP et al Inhaled 1, 8-cineole reduces inflammatory parameters in airways of ovalbumin-challenged Guinea Pigs. Basic Clin Pharmacol Toxicol. 2011 108: 34–39. doi: 10.1111/j.1742-7843.2010.00622.x 20722639 10.1111/j.1742-7843.2010.00622.x

[24] Serafino A, Sinibaldi Vallebona P, Andreola F, Zonfrillo M, Mercuri L, Federici M, et al Stimulatory effect of *Eucalyptus* essential oil on innate cell-mediated immune response. BMC Immunol 2008, 9:17 doi: 10.1186/1471-2172-9-17 18423004 10.1186/1471-2172-9-17 PMC2374764

[25] Rantzsch U, Vacca G, Duck R, Gillissen A. Anti-inflammatory effects of myrtol standardized and other essential oils on alveolar macrophages from patients with chronic obstructive pulmonary disease. Eur J Med Res. 2009; 14(Suppl. IV): 205–209. 20156758 10.1186/2047-783X-14-S4-205 PMC3521325

[26] Subramani R, Narayanasamy M, Feussner KD. Plant-derived antimicrobials to fight against multi-drug-resistant human pathogens. 3 Biotech. 2017; 7(3): 172 doi: 10.1007/s13205-017-0848-9 28660459 10.1007/s13205-017-0848-9 PMC5489455

[27] Medina E, Pieper DH. Tackling threats and future problems of multidrug-resistant bacteria. Curr Top Microbiol Immunol. 2016; 398: 3–33. doi: 10.1007/82_2016_492 27406189 10.1007/82_2016_492

[28] Pérez-Rosés R, Risco E, Vila R, Peñalver P, Cañigueral S. Effect of Some Essential Oils on Phagocytosis and Complement System Activity. J Agric Food Chem. 2015; 63(5):1496–504. doi: 10.1021/jf504761m 25599399 10.1021/jf504761m

[29] Bode JG, Ehlting C, Haussinger D. The macrophage response towards LPS and its control through the p38(MAPK)-STAT3 axis. Cell Signal. 2012; 24: 1185–1194. doi: 10.1016/j.cellsig.2012.01.018 22330073 10.1016/j.cellsig.2012.01.018

[30] Kim YH, Choi KH, Park JW, Kwon TK. LY294002 inhibits LPS-induced NO production through a inhibition of NF-κB activation: independent mechanism of phosphatidylinositol 3-kinase. Immunol letters. 2005; 99(1): 45–50. 10.1016/j.imlet.2004.12.007 15894110

[31] Luyendyk JP, Schabbauer GA, Tencati M, Holscher T, Pawlinski R, Mackman N. Genetic analysis of the role of the PI3K-Akt pathway in lipopolysaccharide-induced cytokine and tissue factor gene expression in monocytes/macrophages. J Immunol. 2008; 180(6): 4218–4226. 18322234 10.4049/jimmunol.180.6.4218 PMC2834303

[32] Bouchon A, Facchetti F, Weigand MA, Colonna M. TREM-1 amplifies inflammation and is a crucial mediator of septic shock. Nature. 2001; 410(6832): 1103–1107. doi: 10.1038/35074114 11323674 10.1038/35074114

[33] Murakami Y, Kohsaka H, Kitasato H, Akahoshi T. Lipopolysaccharide-induced up-regulation of Triggering Receptor Expressed on Myeloid Cells-1 expression on macrophages is regulated by endogenous prostaglandin E2. J Immunol. 2007; 178(2): 1144–1150. 17202378 10.4049/jimmunol.178.2.1144

[34] Gurung P, Li B, Subbarao Malireddi RK, Lamkanfi M, Geige TL, Kanneganti TD. Chronic TLR stimulation controls NLRP3 inflammasome activation through IL-10 mediated regulation of NLRP3 expression and Caspase-8 activation. Sci Rep. 2015; 5: 14488 doi: 10.1038/srep14488 26412089 10.1038/srep14488 PMC4585974

[35] Baroja-Mazo A, Martín-Sánchez F, Gomez AI, Martínez CM, Amores-Iniesta J, Compan V, et al The NLRP3 inflammasome is released as a particulate danger signal that amplifies the inflammatory response. Nat Immunol. 2014; 15:738–748. doi: 10.1038/ni.2919 24952504 10.1038/ni.2919

[36] Guo H, Callaway JB, Jenny Ting PY. Inflammasomes: mechanism of action, role in disease, and therapeutics. Nat med. 2015; 21(7): 671–887. 10.1038/nm.3893 PMC4519035 26121197

[37] Lloberas J, Valverde-Estrella L, Tur J, Vico T, Celada A. Mitogen-activated protein kinases and mitogen kinase phosphatase 1: A critical interplay in macrophage biology. Front Mol Biosci. 2016; 28(3): 28 doi: 10.3389/fmolb.2016.00028 27446931 10.3389/fmolb.2016.00028 PMC4923182

[38] Tucsek Z, Radnai B, Racz B, Debreceni B, Priber JK, Dolowschiak T, et al Suppressing LPS- induced early signal transduction in macrophages by a polyphenol degradation product: a critical role of MKP-1. J Leukoc Biol. 2011; 89(1): 105–11. doi: 10.1189/jlb.0610355 20884647 10.1189/jlb.0610355

[39] Khan NM, Haseeb A, Ansari MY, Devarapalli P, Sara HS, Haqqi TM. Wogonin, a plant derived small molecule, exerts potent anti-inflammatory and chondroprotective effects through the activation of ROS/ERK/Nrf2 signaling pathways in human Osteoarthritis chondrocytes. Free Radic Biol Med. 2017; 106: 288–301. doi: 10.1016/j.freeradbiomed.2017.02.041 28237856 10.1016/j.freeradbiomed.2017.02.041 PMC5490997

[40] Linda M, Hayes A, Caprnda M, Petrovic D, Rodrigo L, Kruzliak P, et al Inducible nitric oxide synthase: Good or bad? Biomed Pharmacother. 2017; 93: 370–375. doi: 10.1016/j.biopha.2017.06.036 28651238 10.1016/j.biopha.2017.06.036

[41] Gochman E, Mahajna J, Shenzer P, Dahan A, Blatt A, Elyakim R, et al The expression of iNOS and nitrotyrosine in colitis and colon cancer in humans, Acta Histochem. 2012; 114: 827–835. doi: 10.1016/j.acthis.2012.02.004 22417974 10.1016/j.acthis.2012.02.004

[42] Zhao X, Kong J, Zhao Y, Wang X, Bu P, Zhang C, et al Gene silencing of TACE enhances plaque stability and improves vascular remodeling in a rabbit model of atherosclerosis. Sci. Rep. 2015; 5: 1–13. 10.1038/srep17939 PMC4677302 26655882

[43] Atochina-Vasserman EN, Beers MF, Kadire H, Tomer Y, Inch A, Scott P, et al Selective inhibition of inducible NO synthase activity in vivo reverses inflammatory abnormalities in Surfactant Protein D-Deficient mice. J. Immunol. 2007; 179: 8090–8097. 18056350 10.4049/jimmunol.179.12.8090 PMC4009628

[44] Chi H, Flavell RA. Acetylation of MKP-1 and the control of inflammation. Sci Signal. 2008; 1(41): pe44 doi: 10.1126/scisignal.141pe44 18922786 10.1126/scisignal.141pe44 PMC2613485

[45] Chi H, Barry SP, Roth RJ, Wu JJ, Jones EA, Bennett AM, et al Dynamic regulation of pro- and anti-inflammatory cytokines by MAPK phosphatase 1 (MKP-1) in innate immune responses. Proc Natl Acad Sci U S A. 2006; 103(7): 2274–2279. doi: 10.1073/pnas.0510965103 16461893 10.1073/pnas.0510965103 PMC1413743

[46] Hammer M, Mages J, Dietrich H, Servatius A, Howells N, Cato AC, et al Dual specificity phosphatase 1 (DUSP1) regulates a subset of LPS-induced genes and protects mice from lethal endotoxin shock. J Exp Med. 2006; 203(1): 15–20. doi: 10.1084/jem.20051753 16380512 10.1084/jem.20051753 PMC2118077

