# Supplementary material for: Suppression of inflammatory and infection responses in lung macrophages by eucalyptus oil and its constituent 1,8-cineole: Role of pattern recognition receptors TREM-1 and NLRP3, the MAP kinase regulator MKP-1, and NFκB
Source: PLoS One. 2017 Nov 15;12(11):e0188232. doi: 10.1371/journal.pone.0188232 (PMC5687727; doi:10.1371/journal.pone.0188232)
Supplement: S1 File — For Fig 5: (A-D) EO pre-treatment blots; (E-H) Cin pre-treatment blots for p38, SAPK/ JNK, ERK1/2, and NF-kB. MAPKs, respectively. Lane M represent protein molecular weight ladder, lanes1-4 represent vehicle control (VC), EO-only, LPS-only and EO+LPS for EO pre-treatment group and VC, Cin, LPS and Cin +LPS for Cin pre-treatment group, respectively. For Fig 6: Lane M represent protein molecular weight ladder, lanes1-6 represent VC, EO, Cin, LPS, and EO+LPS, Cin +LPS, respectively. (PDF) [file pone.0188232.s001.pdf]

Supplementary information: On figures 5 and 6 western blots

Figure 5 Panel A

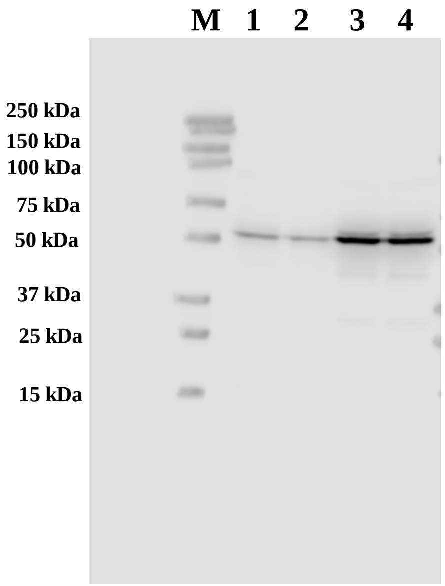

Blot _Phos p38 (EO)

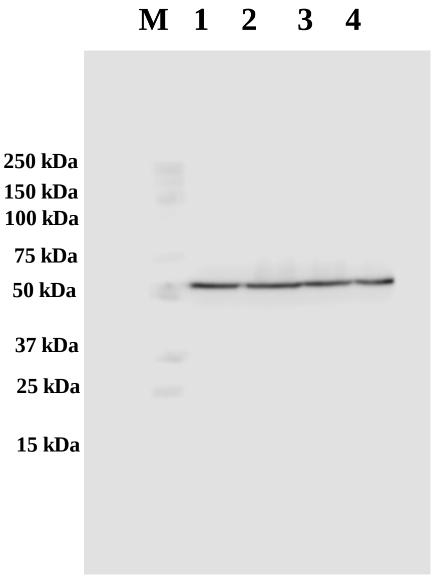

Blot_ Total p38 (EO)

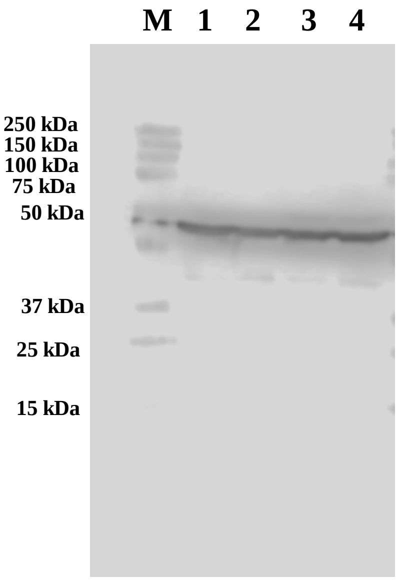

Blot_ β-actin (EO)

Figure 5 Panel B

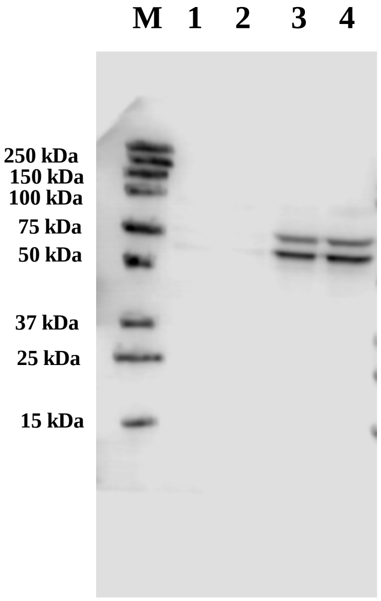

Blot_ Phos JNK1/2 (EO)

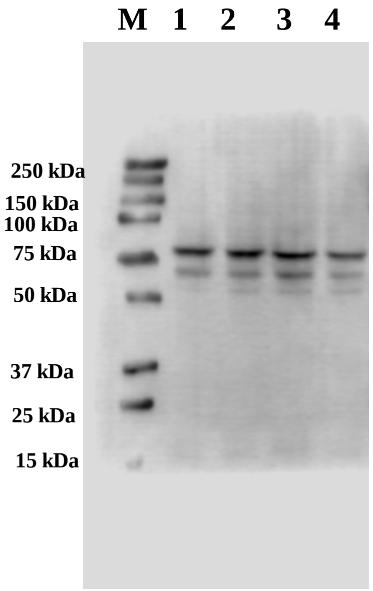

Blot_ Total JNK1/2 (EO)

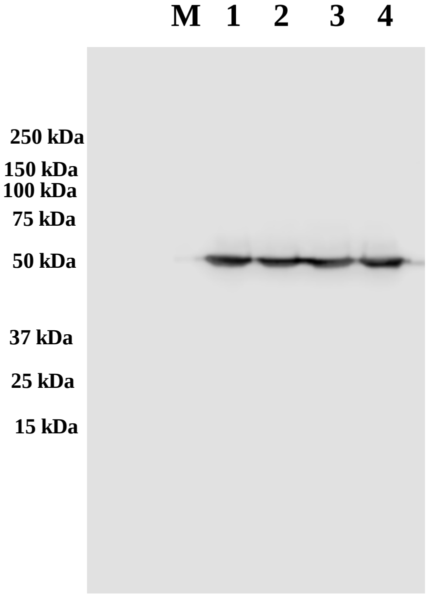

Blot_ β-actin (EO)

Figure 5 Panel C

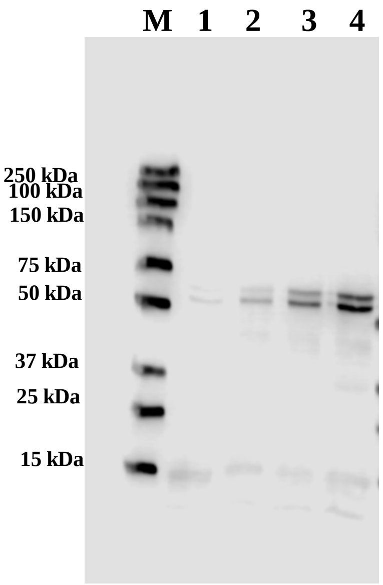

Blot_ Phos ERK1/2 (EO)

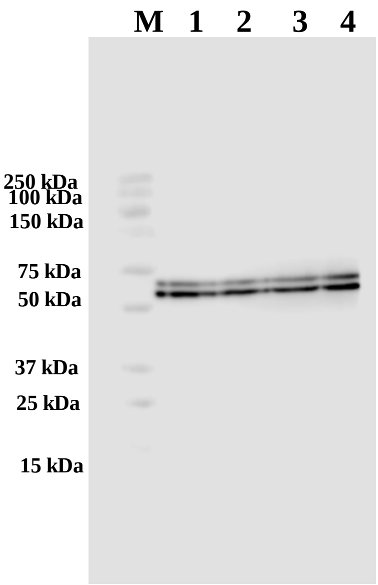

Blot_ Total ERK1/2 (EO)

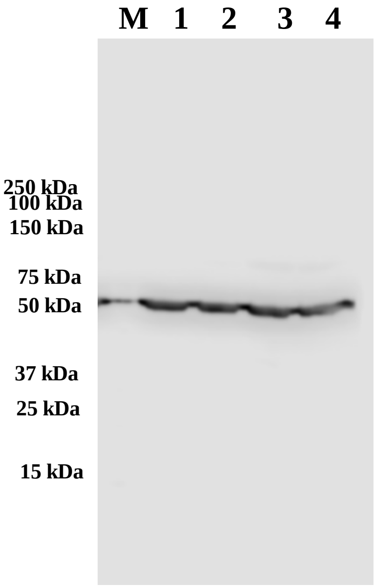

Blot_ β-actin (EO)

Figure 5 Panel D

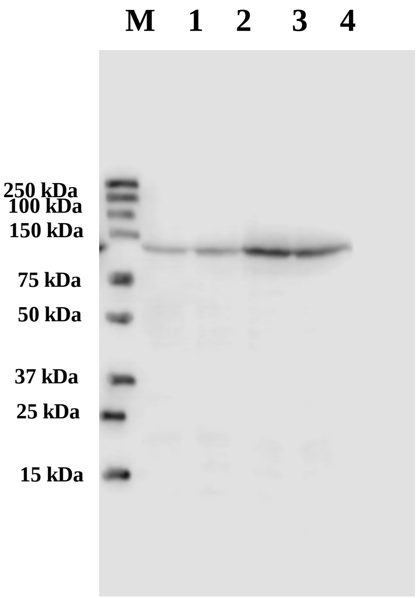

Blot_ Phos NFκB (EO)

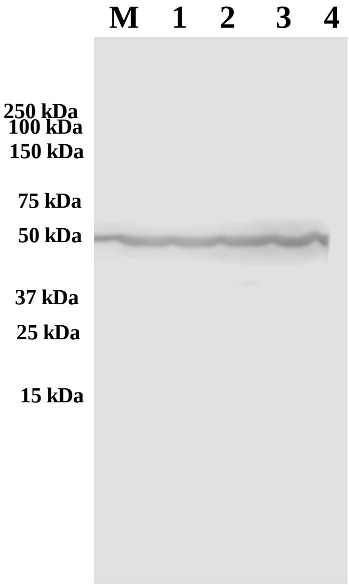

Blot_ β-actin (EO)

Figure 5 Panel E

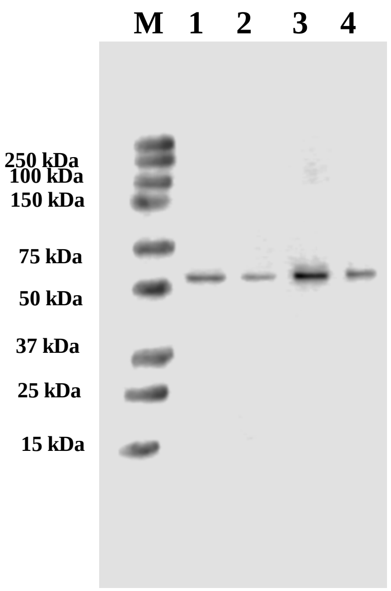

Blot_ Phos p38 (Cineole)

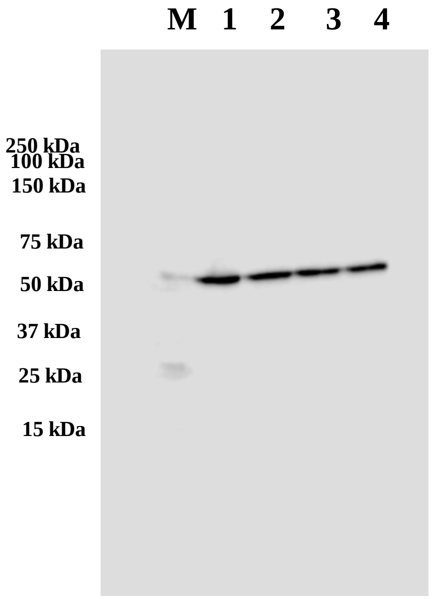

Blot_ Total p38 (Cineole)

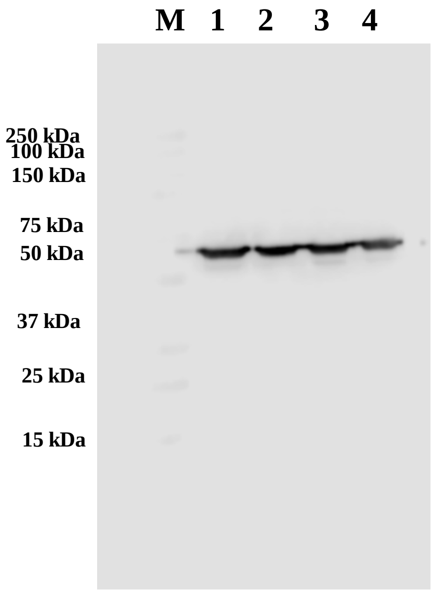

Blot_ B-actin (Cineole)

Figure 5 Panel F

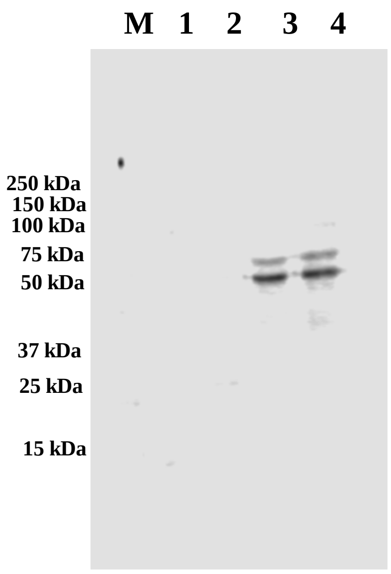

Blot_ Phos JNK1/2 (Cineole)

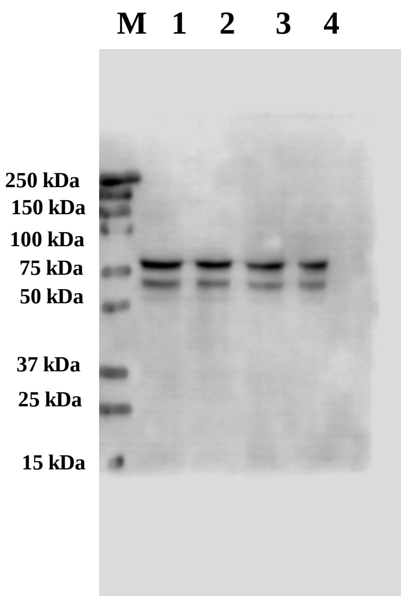

Blot _Total JNK1/2 (Cineole)

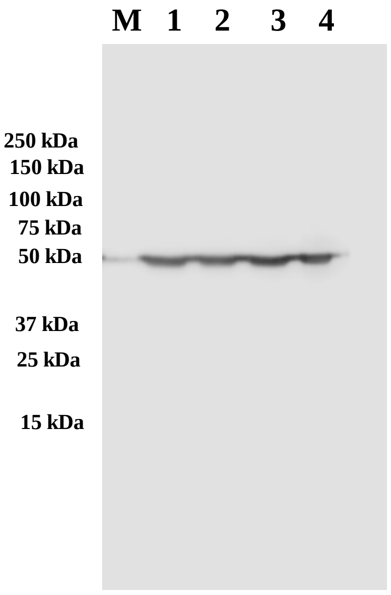

Blot_ B-actin (Cineole)

Figure 5 Panel G

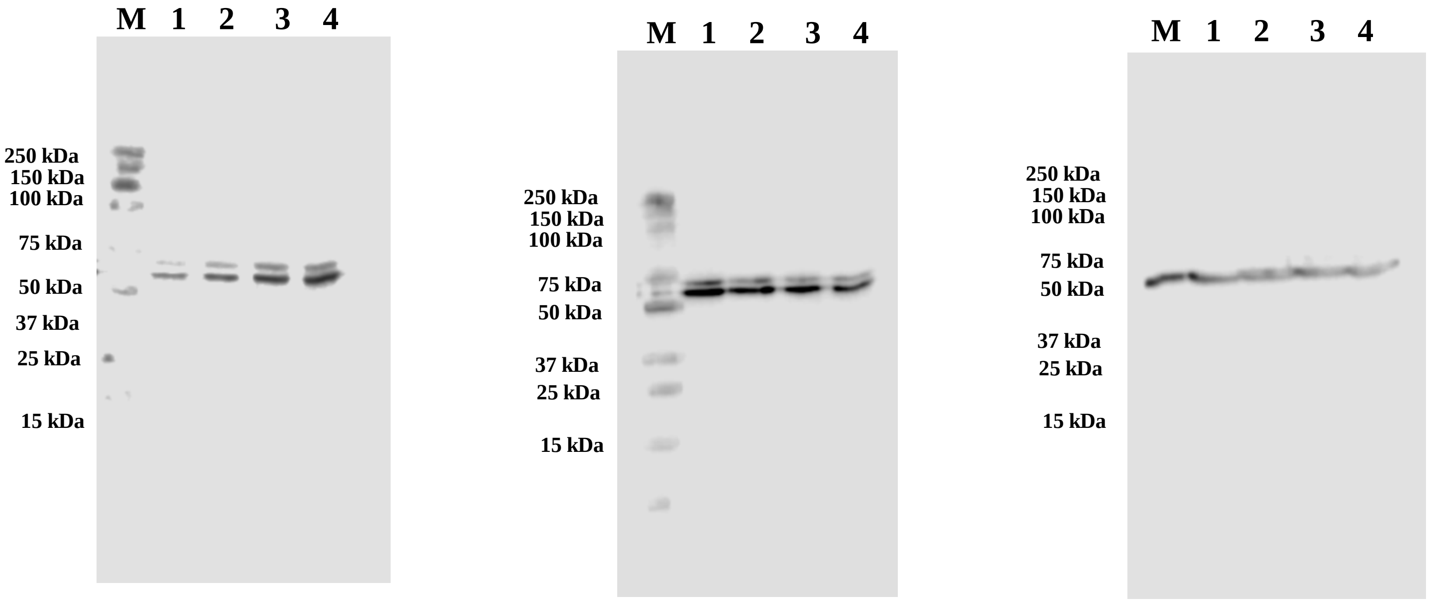

Blot_ Phos ERK1/2 (Cineole) Blot_ Total ERK1/2 (Cineole) Blot_ β-actin (Cineole)

Figure 5 Panel H

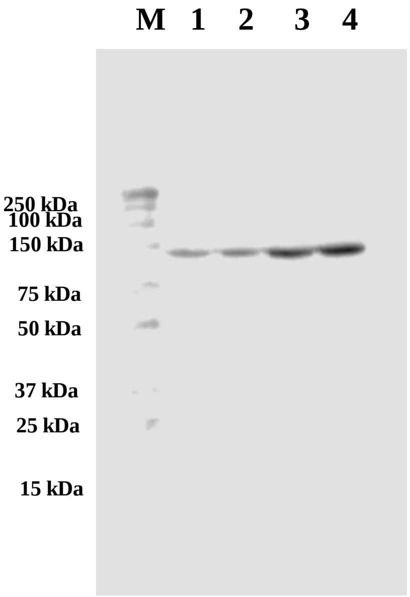

Blot_ Phos NFκB (Cineole)

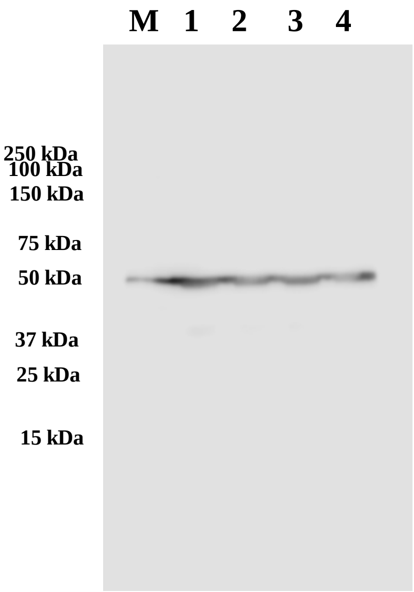

Blot_ β-actin (Cineole)

Figure 6

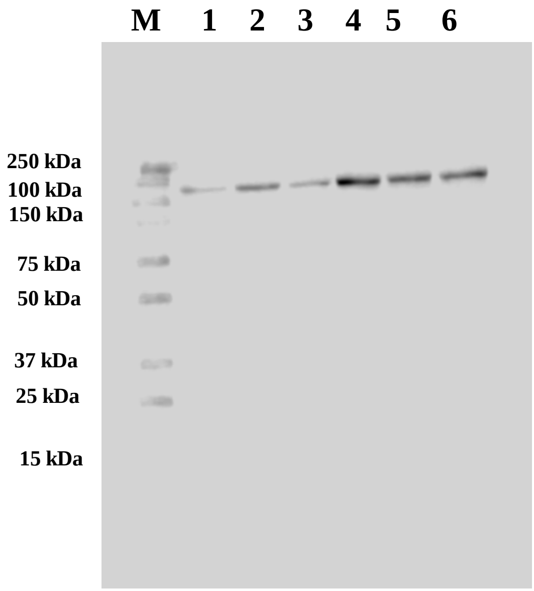

Blot_ NLRP3

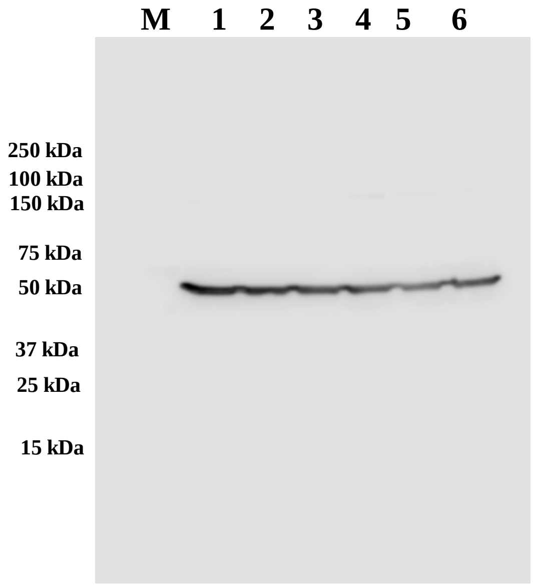

Blot_ β- actin NLRP3
